# Prevalence of human papillomavirus (HPV) in Brazil: A systematic review and meta-analysis

**DOI:** 10.1371/journal.pone.0229154

**Published:** 2020-02-21

**Authors:** Verônica Colpani, Frederico Soares Falcetta, Augusto Bacelo Bidinotto, Natália Luiza Kops, Maicon Falavigna, Luciano Serpa Hammes, Adele Schwartz Benzaken, Ana Goretti Kalume Maranhão, Carla Magda Allan S. Domingues, Eliana Márcia Wendland

**Affiliations:** 1 Hospital Moinhos de Vento, Porto Alegre, Rio Grande do Sul, Brazil; 2 Tropical Medicine Foundation Heitor Vieira Dourado, Manaus, Amazonas, Brazil; 3 Aids Health Care Foundation, Manaus, Amazonas, Brazil; 4 National Immunization Program, Ministry of Health, Brasilia, Federal District, Brazil; 5 Department of Community Health, Federal University of Health Science of Porto Alegre, Porto Alegre, Rio Grande do Sul, Brazil; Universidade Estadual de Maringa, BRAZIL

## Abstract

**Objectives:**

This study aimed to estimate the prevalence of genital, anal and oral HPV infection in Brazil through systematic review and meta-analysis.

**Methods:**

We searched EMBASE, LILACS, MEDLINE, Web of Science and SciELO from inception to December 2018. Original research articles that assessed the prevalence of genital (i.e., cervical, penile), anal and oral HPV infection in Brazil were selected in pairs by independent authors. No sex, age, HPV vaccination, language or date restrictions were applied. HPV prevalence was estimated and stratified according to risk factors population and by geographic area throughout the country. The study prevalence was pooled using a random effects model. Analysis was performed using R (version 3.5.2), packages meta version 4.9–4 and metaphor 2.0–0. This review is registered on PROSPERO under protocol number CRD42016032751.

**Results:**

We identified 3,351 references. After the screening process, 139 of them were eligible for this systematic review (57,513 total participants). Prevalence of cervical HPV was 25.41% (95% CI 22.71–28.32). Additionally, prevalence was 36.21% (95% CI 23.40, 51.33) in the penile region, 25.68% (95%CI 14.64, 41.04) in the anal region, and 11.89% (95%CI 6.26, 21.43) in the oral region. Subgroup analysis showed prevalence in each anatomic site was higher in high-risk populations.

**Conclusion:**

The prevalence of HPV is high in the Brazilian population and varies by population risk and anatomic body site, with lower rates in the oral cavity compared to that in the cervical, penile and anal region. Studies on HPV have primarily been developed to evaluate infection and cancer in the cervical region. There is a profound lack of HPV data in many geographic regions of Brazil and for different anatomic sites.

## Introduction

Human papillomavirus (HPV) is a DNA virus from the Papillomaviridae family, comprising over 170 identified types [1]. HPV may infect skin and mucosal membranes among different anatomic sites such as the anogenital region and oral cavity [2]. The global prevalence of HPV infection is approximately 12% with substantial regional variation [3]. In Brazil, previous studies reported a prevalence of genital HPV varying between 10.4 and 72%, depending of sex [4,5], and a prevalence of 6.2% of oropharyngeal HPV [6].

HPV is the most common sexually transmitted infection, with sufficient evidence of its carcinogenic effects at different sites [1,4]. Worldwide, HPV is responsible for 5.1% of the burden of cancer [6] and is present in nearly 100% of cervical tumors, 88% of anal tumors and 50% of penile tumors [4]. In head and neck squamous cell carcinomas, HPV-16 is the most commonly found genotype, while overall HPV prevalence in these lesions is 26% [7].

HPV prevalence varies widely between genders as well as among anatomic sites [8]. The worldwide HPV prevalence varies according to the population analyzed and the status of economic development of the region [4]. Since Brazil is a country with continental proportions and significant socioeconomic diversity, the epidemiology of HPV infection within Brazil might also be impacted by these variables.

The HPV National Vaccination Program in Brazil started in 2014, and it will take time to determine the effect of vaccination on the HPV rates [9]. Understanding the HPV prevalence prior to vaccination will be useful to monitor changes related to the vaccine as well as changes in the distribution of HPV among different areas in Brazil. In addition, obtaining data that could help us make inferences about the HPV-infected population at a national level would facilitate the decision-making process and guide campaigns and public investments in the sexually active population in Brazil.

Therefore, the aim of this systematic review and meta-analysis was to assess the prevalence of genital, anal and oral HPV infection in Brazil to provide support for the Ministry of Health to establish health policies and programs.

## Materials and methods

This review has been registered on PROSPERO under protocol number CRD42016032751. This protocol has also been published in a peer reviewed open access journal [10].

### Search

We performed the search of the following databases through December 28, 2018 [10]: EMBASE, LILACS, MEDLINE, Web of Science and SciELO using terms as “human papillomavirus”, “HPV”, “prevalence” and “Brazil” (S1 Appendix); we also scanned the reference lists of identified publications for additional studies. Brazilian specialists in the field were contacted to identify unpublished and ongoing studies and we used the website “bancodeteses.capes.gov.br” to identify any thesis in the area, and websites such as the Grey Literature Report (www.greylit.org). Data from conference proceedings were included (even without an author response) if the abstract provided enough information to assess its eligibility and to extract at least the overall prevalence and number of participants.

### Study selection and data extraction

The inclusion criteria for articles were as follows: (1) randomized controlled trials, cohort studies, cross-sectional studies, or prevalence studies; (2) studies evaluating the prevalence of genital (cervical and penile), anal, and oral HPV infection in Brazil; and (3) HPV assessment via polymerase chain reaction (PCR), hybrid capture or any well-described genotyping methodology. We excluded studies evaluating only pregnant women or HIV-positive participants as well as studies that analyzed material such as blood, sperm and urine. No sex, age, HPV vaccination, language or date restrictions were applied.

The evaluation of titles and abstracts was conducted by either VC and ABB or NK and FSF, always in pairs, and using standardized forms. Discrepancies were solved through consensus. We collected the following data: publication title, authors, publication year, study design, population characteristics (e.g., high-risk population, number of participants, gender, age, and geographic area in Brazil), the number of HPV-positive and HPV-negative, HPV type, HPV detection and genotyping methodology. For cohort and randomized studies, we extracted only the baseline characteristics of the population. For studies with no data regarding the geographic area where the samples were collected, we contacted the authors to obtain this information. If duplicate studies were discovered, either the study published earlier or the study that provided more information was included. An email was sent to the authors of the eligible duplicate studies to obtain more details about these articles.

We stratified the study population according to risk factors. The population samples/community were defined as low-risk when including asymptomatic healthy individuals who were enrolled in a cancer screening program, attended primary care clinics or did not have any clinical lesions, whereas the high-risk population was defined as partners of women positive for a lesion (HPV or cervical intraepithelial neoplasia; CIN), women with cervical lesion or recruited from a sexual health clinic or key populations (intravenous drug users, men who have sex with men, transgender persons, sex workers and prisoners).

The included studies were assessed for quality using an adapted version of the NIH’s ‘Quality Assessment Tool for Observational Cohort and Cross-Sectional Studies [11]. Reviewers classified the methodological quality of each study and its risk of bias as good, unclear or poor, according to potentials flaws in the study methods. The overall quality of evidence was assessed using Grades of Recommendation, Assessment, Development, and Evaluation (GRADE), a methodology that rates the confidence in estimates of a body of evidence considering the following domains: risk of bias, imprecision, inconsistency, indirectness, and publication bias. We followed the approach proposed for the assessment of baseline risk in prognostic studies and we applied the GRADE framework developed for incidence, as there is no formal procedure for the assessment of certainty of evidence in estimates in the context of prevalence studies [12].

### Statistical analysis

Random effects models were used to calculate the pooled prevalence measures and the corresponding 95% confidence intervals (CI). The heterogeneity of the findings was assessed using both the *Cochran’s Q* test and the *I*^2^ statistic. Sensitivity analysis was conducted by meta-regression analyses to investigate the effect of the genotyping method. We performed subgroup analyses for the population risk factor (low- or high-risk), geographic area (i.e., Brazilian region), anatomic site (i.e., cervical, penile, anal and oral regions), high-risk HPV (HR-HPV) type, and presence of HPV-16 and 18. Studies in which the HPV type (high- or low-risk) was either not assessed or not reported were excluded from the HPV type-specific analyses. We also estimated prediction intervals to provide a range of expected HPV prevalence among Brazilian population [13]. Publication bias across the studies was evaluated using funnel plots and Begg’s tests. Analysis was performed using R (version 3.6.1), packages meta version 4.9–7 and metafor 2.0–0.

## Results

### Descriptive overview of included studies

**Fig 1:**
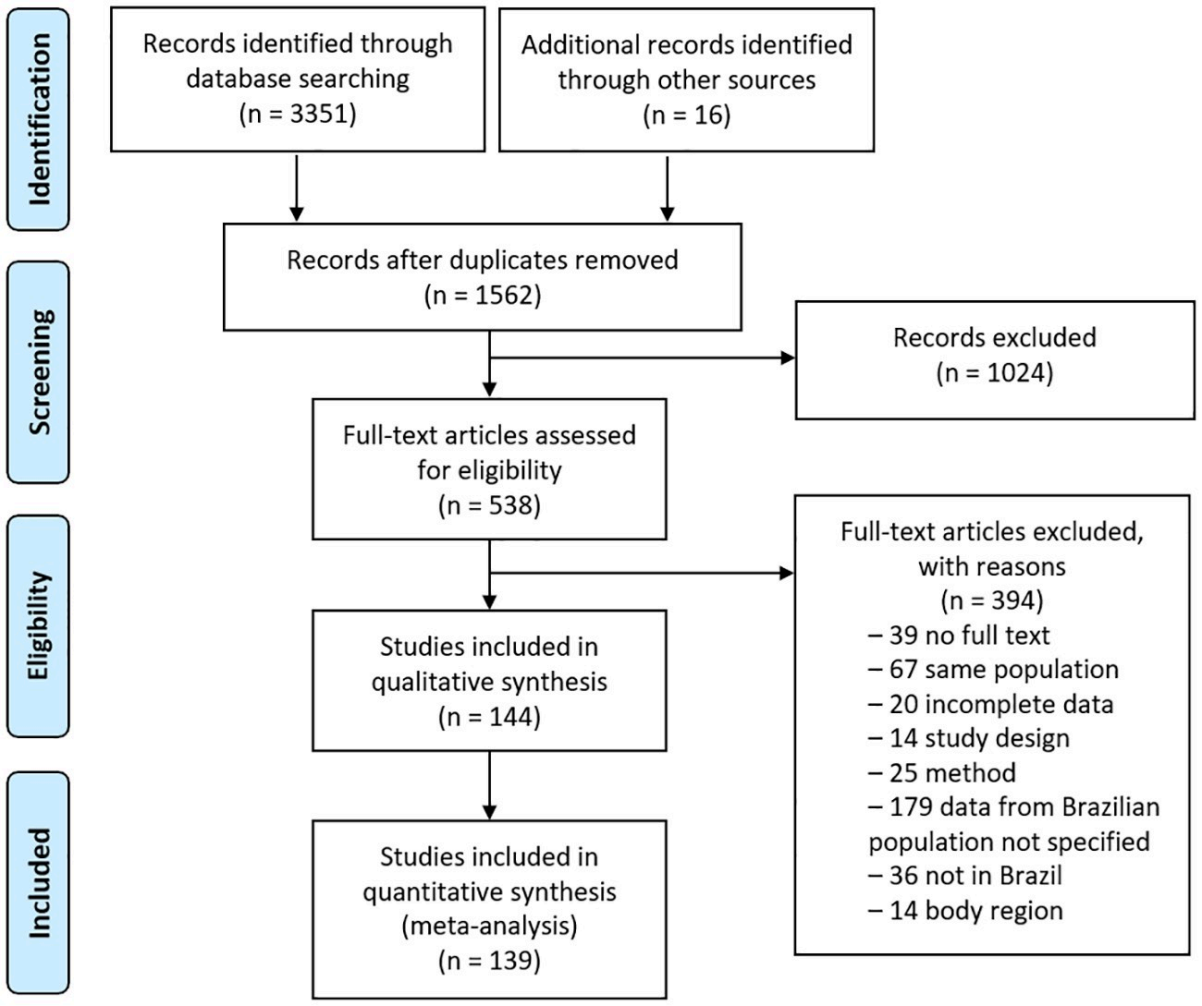
PRISMA flow diagram for the selection of included studies.

We identified 3,351 abstracts from the selected databases, and 16 additional studies were included from the listed references of the identified manuscripts as well as other sources. From the 1,562 unique citations, 1,024 were excluded, and the full text of the remaining 538 publications were read. We excluded 394 articles in the subsequent full-text assessments for reasons such as absence of full text, same population of articles already included, incomplete data, different designs form the inclusion criteria, different methods from the inclusion criteria, different anatomic site from the inclusion criteria, without specific data from Brazilian population, or without Brazilian population (Fig 1). After this evaluation, 144 articles reporting the prevalence of HPV [14–157] remained for qualitative synthesis and 139 articles remained for the quantitative analysis (meta-analysis).

A summary of the studies characteristics is presented in Table 1. The included studies evaluated 57,513 participants from 139 studies that provided data for quantitative analysis. There were 105 studies that evaluated the presence of HPV in the cervical region, 12 in the penile region, 7 in the anal region and 20 in the oral region. Regarding the diagnostic method, approximately 86.8% of articles used polymerase chain reaction. Most of the studies (42%) originated from the Southeast region (S1 Table).

**Table 1:** Meta-analysis of the prevalence of HPV by geographic region in Brazil.

Region	N studies	N patients	Method	Prevalence (CI 95%)	I^2^ (%)
**Cervical region**					
South	27	10,661	22 PCR / 3 HC / 2 undescribed	21.59% (18.34, 25.23)	94.0
Southeast	37	26,077	30 PCR / 7 HC*	25.65% (20.70, 31.33)	99.0
North	14	4,787	PCR	20.40% (17.20, 28.63)	95.0
Northeast	15	5,574	13 PCR / 2 HC	32.82% (22.47, 45.15)	99.0
Central-West	8	1,558	7 PCR / 1 HC	31.89% (24.91, 39.78)	89.0
Undefined	4	2,165	3 PCR / 1 HC	29.14% (25.99, 32.51)	41
**Penile region**					
South	1	99	PCR	54.55% (44.23, 64.59)	
Southeast	8	2,638	PCR	39.61% (22.54, 59.64)	98.0
North					
Northeast	2	129	PCR	25.59% (18.80, 33.81)	0
Central-West					
Undefined	1	90	PCR	18.89% (11.41, 28.51)	
**Anal region**					
South					
Southeast	4	1,148	3 PCR / 1HC	21.22% (8.38, 44.24)	96.0
North	1	42	PCR	26.19% (15.14, 41.38)	-
Northeast	2	82	PCR / undescribed	39.20% (29.21, 50.18)	
Central-West					
Undefined					
**Oral region**					
South	2	596	PCR	1.26% (0.62, 2.55)	0
Southeast	11	1,276	10 PCR / 1HC	8.99% (3.47, 21.35)	94.0
North	1	166	PCR	24.10% (18.20, 31.18)	
Northeast	4	266	PCR	37.56% (10.63, 75.25)	96.0
Central-West	1	65	PCR	6.15% (2.33, 15.28)	
Undefined	1	125	PCR	23.20% (16.63, 31.39)	

CI, Confidence Interval; HC, Hybrid Capture; PCR, Polymerase Chain Reaction.

*1 analysis by ELISA

The majority of the included studies were not population-based and did not have information regarding either the sample size power or the participation rate. The overall quality of evidence for HPV prevalence was rated as very low according to GRADE, mainly because the studies had selection bias, inconsistency and provided indirect evidence. The quality of evidence assessment is shown in S2 Table.

Funnel plots were symmetrical for the cervical HPV prevalence analysis, and Begg’s test was not significant for any region of the body, except for the anal region which could not be assessed by the asymmetry test due to the small number of articles (S1 Fig).

### Prevalence of HPV in cervical samples

**Fig 2:**
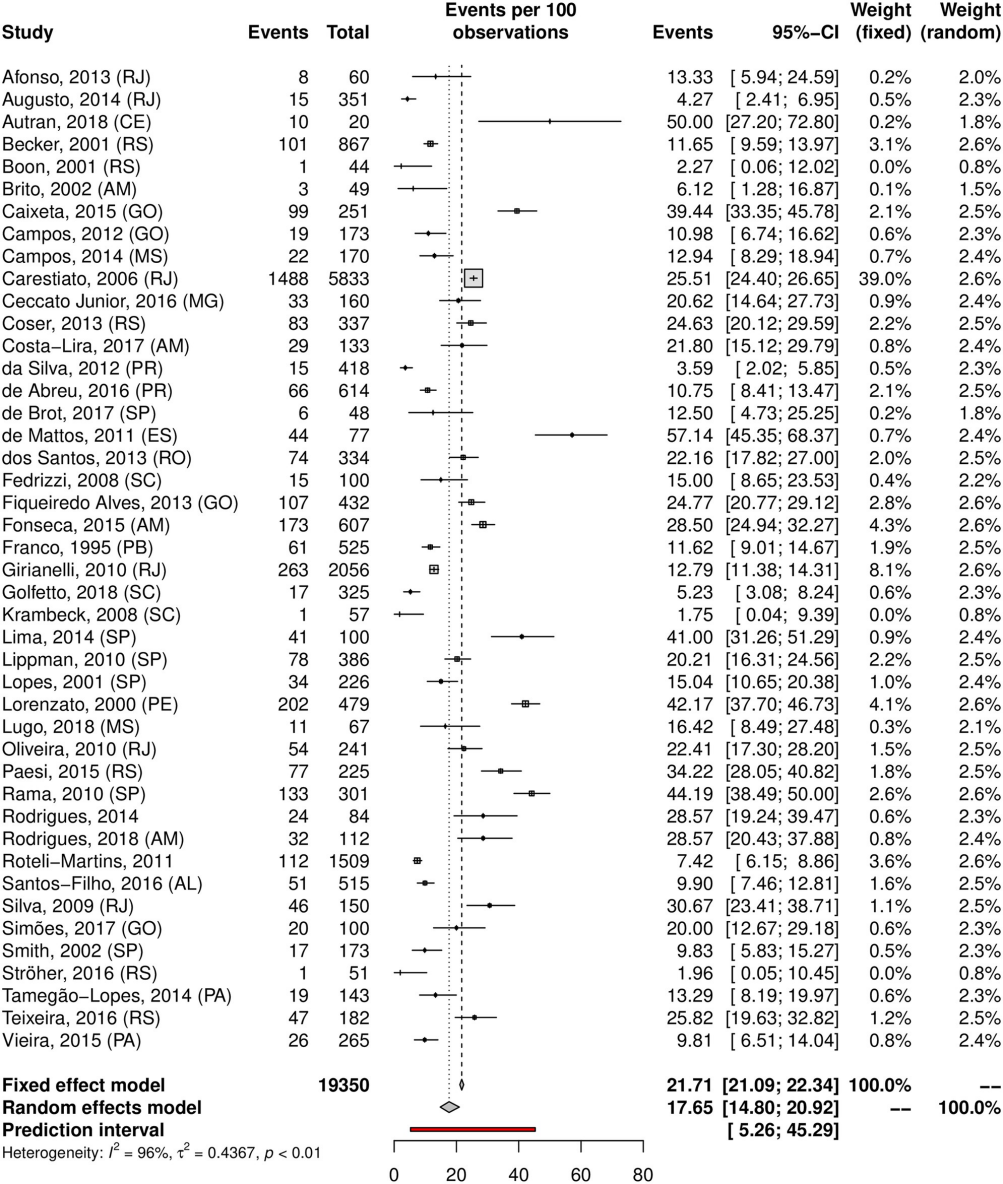
Overall prevalence of cervical infection by high-risk HPV genotypes. Forest plot of a metanalysis of studies reporting prevalence of infection of the cervix by HR-HPV genotypes in Brazil.

**Fig 3:**
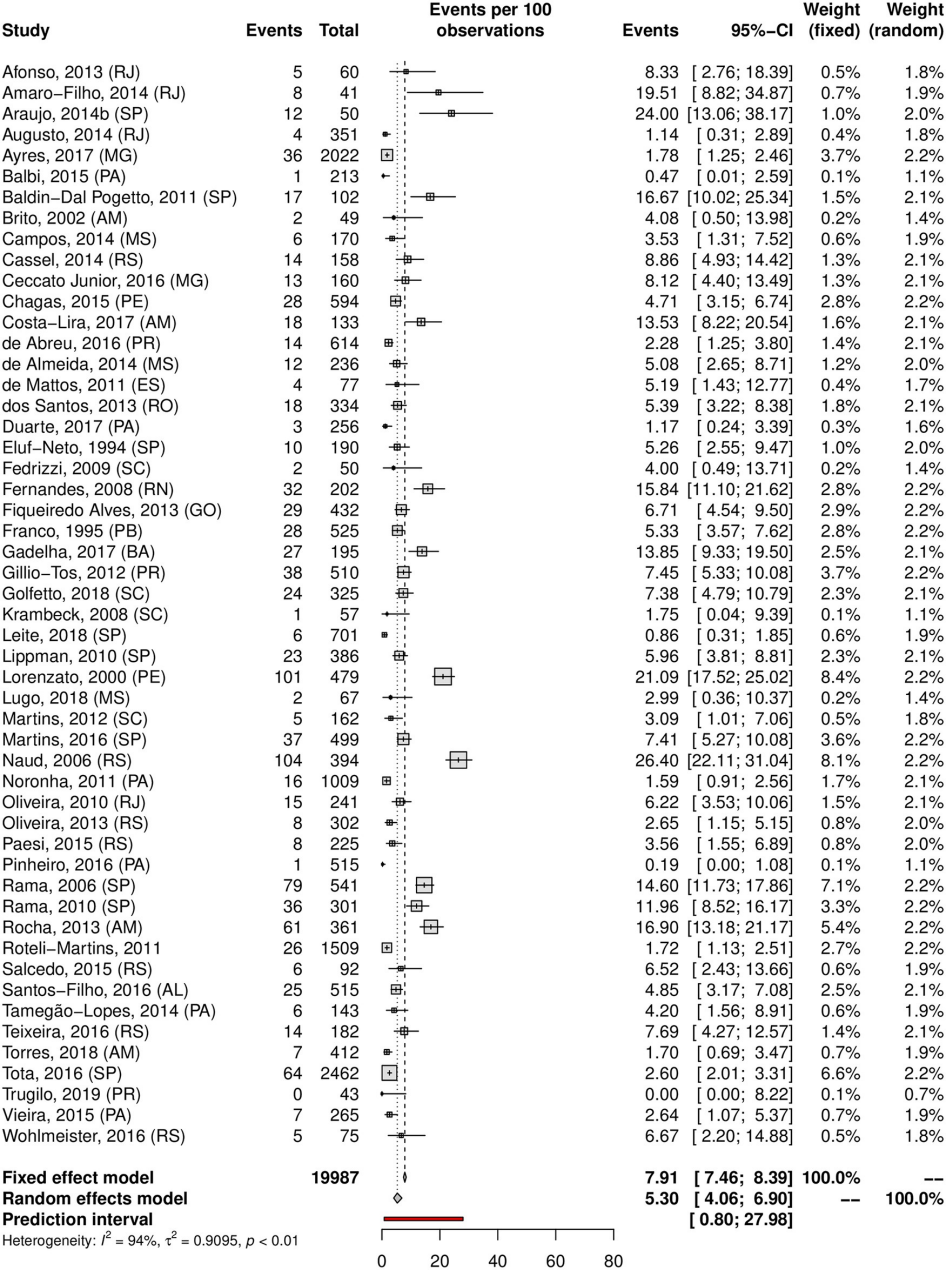
Overall prevalence of cervical infection by HPV-16. Forest plot of a metanalysis of studies reporting prevalence of cervical infection by HPV-16 in Brazil.

**Fig 4:**
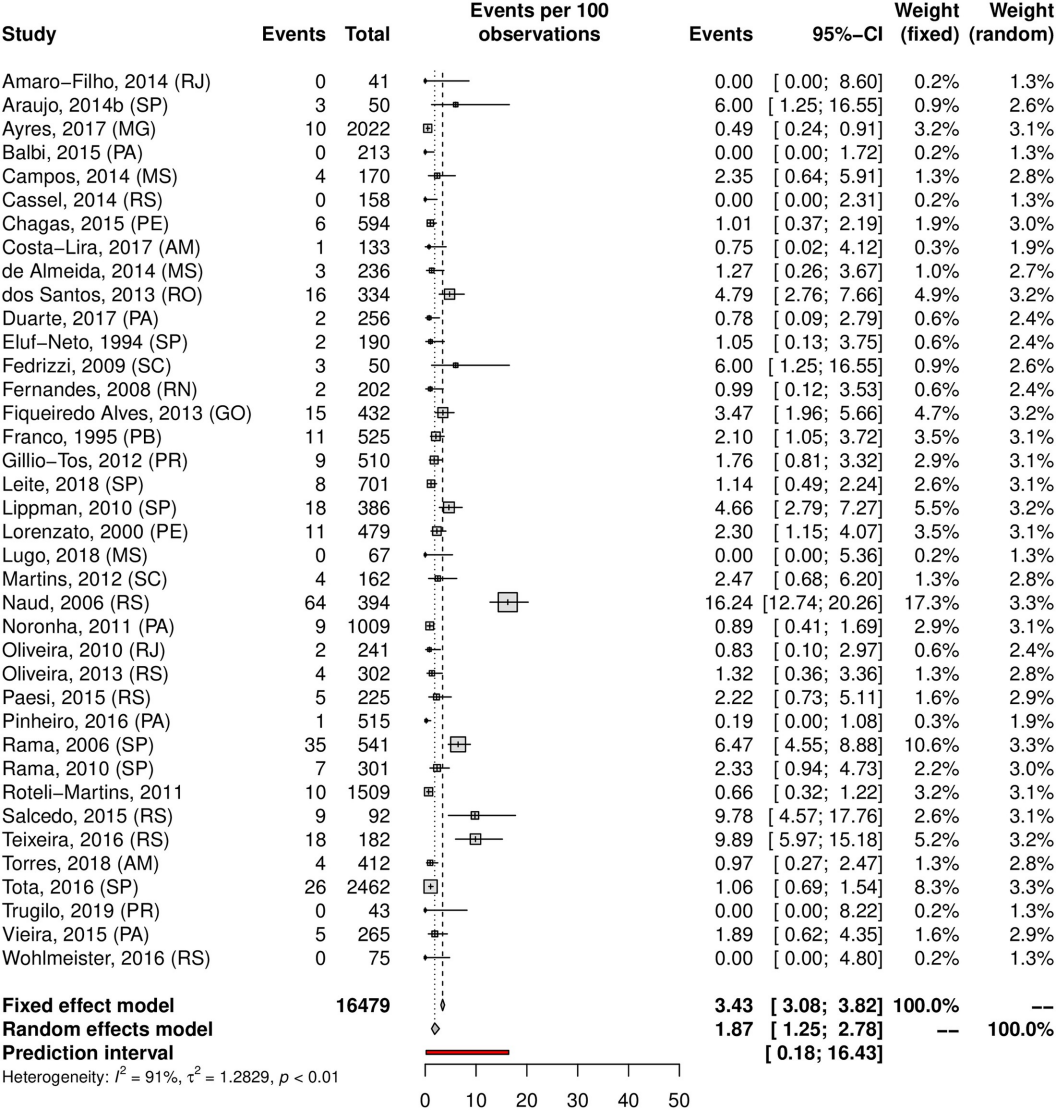
Overall prevalence of cervical infection by HPV-18. Forest plot of a metanalysis of studies reporting prevalence of cervical infection by HPV-18 in Brazil.

The overall prevalence of cervical HPV was 25.41% (95% CI 22.71–28.32; 105 studies; I^2^ = 98%; S2 Fig), and the prevalence of HR-HPV genotypes was 17.65% (95% CI 14.80–20.92; 44 studies; I^2^ = 96%; Fig 2). The prediction interval for HPV prevalence ranged from 7.17 to 60.04%, with 95% confidence. This prediction interval represents the range of expected Brazilian cervical HPV prevalence in 95% of settings. The prevalence of cervical HPV-16 was 5.30% (95% CI, 4.06–6.90; 52 studies; I^2^ = 94%, Fig 3), and HPV-18 was 1.87% (95% CI, 1.25–2.78; 38 studies; I^2^ = 91%, Fig 4).

**Fig 5:**
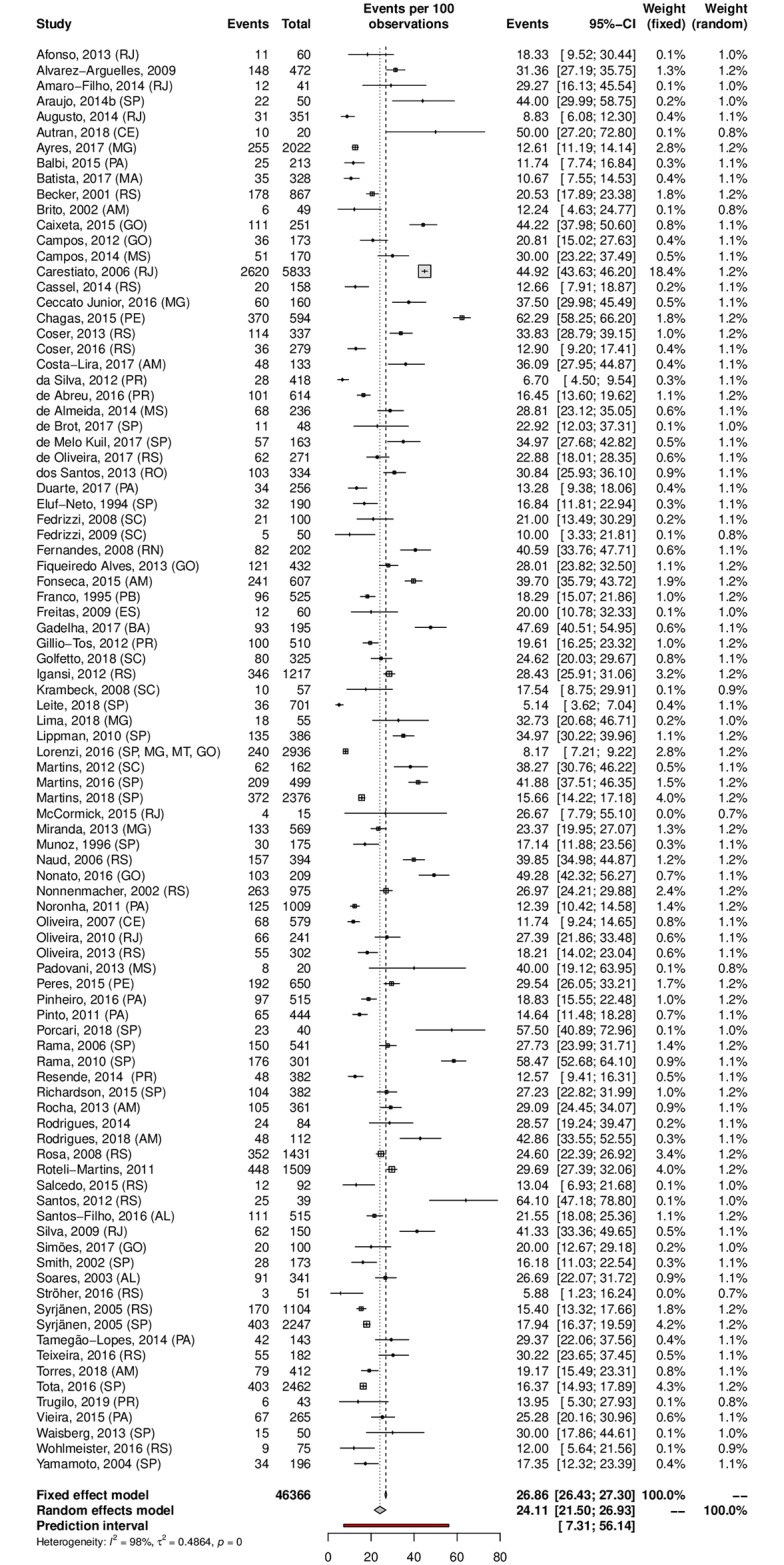
Prevalence of cervical HPV infection in low-risk populations. Forest plot of a metanalysis of studies reporting prevalence of cervical infection by HPV in low-risk populations in Brazil.

**Fig 6:**
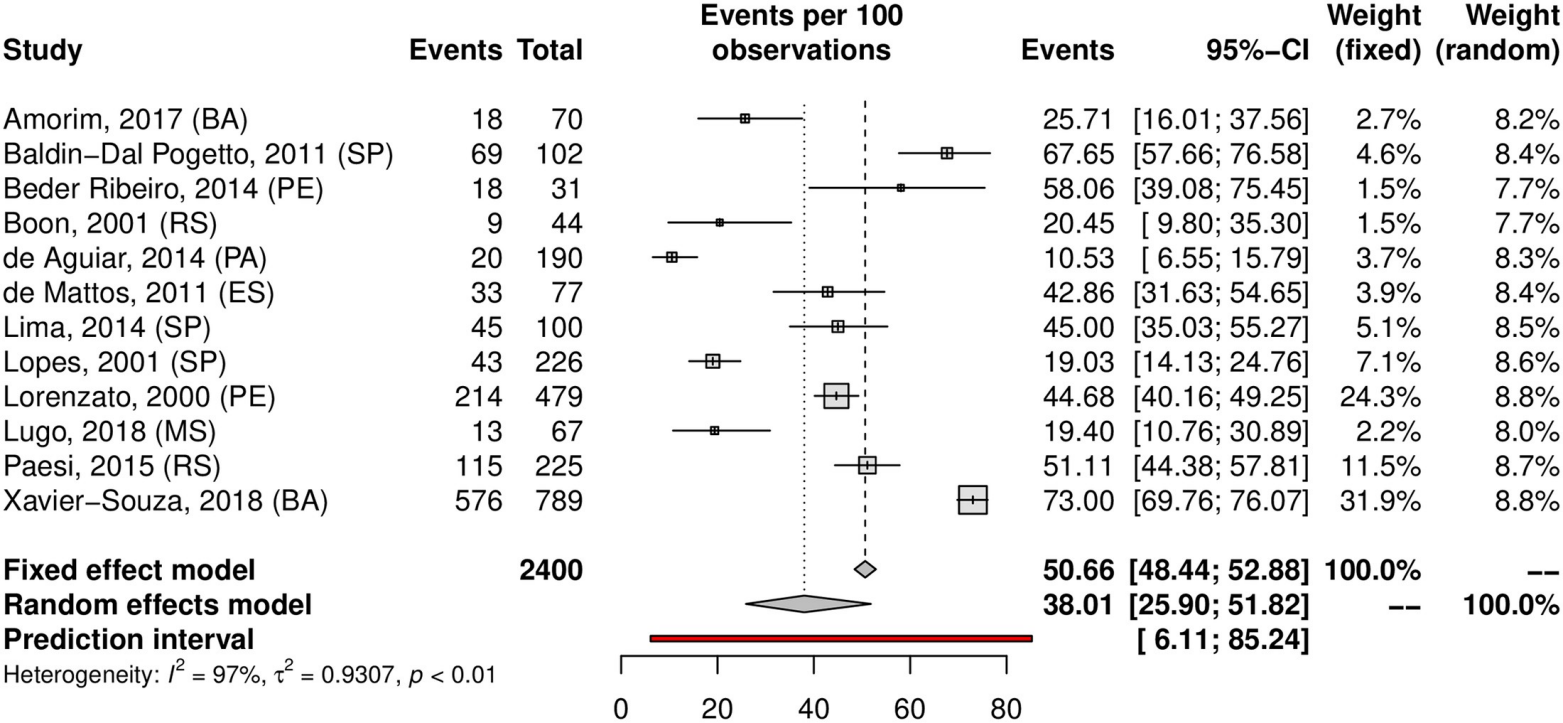
Prevalence of cervical HPV infection in high-risk populations. Forest plot of a metanalysis of studies reporting prevalence of cervical infection by HPV in high-risk populations in Brazil.

**Fig 7:**
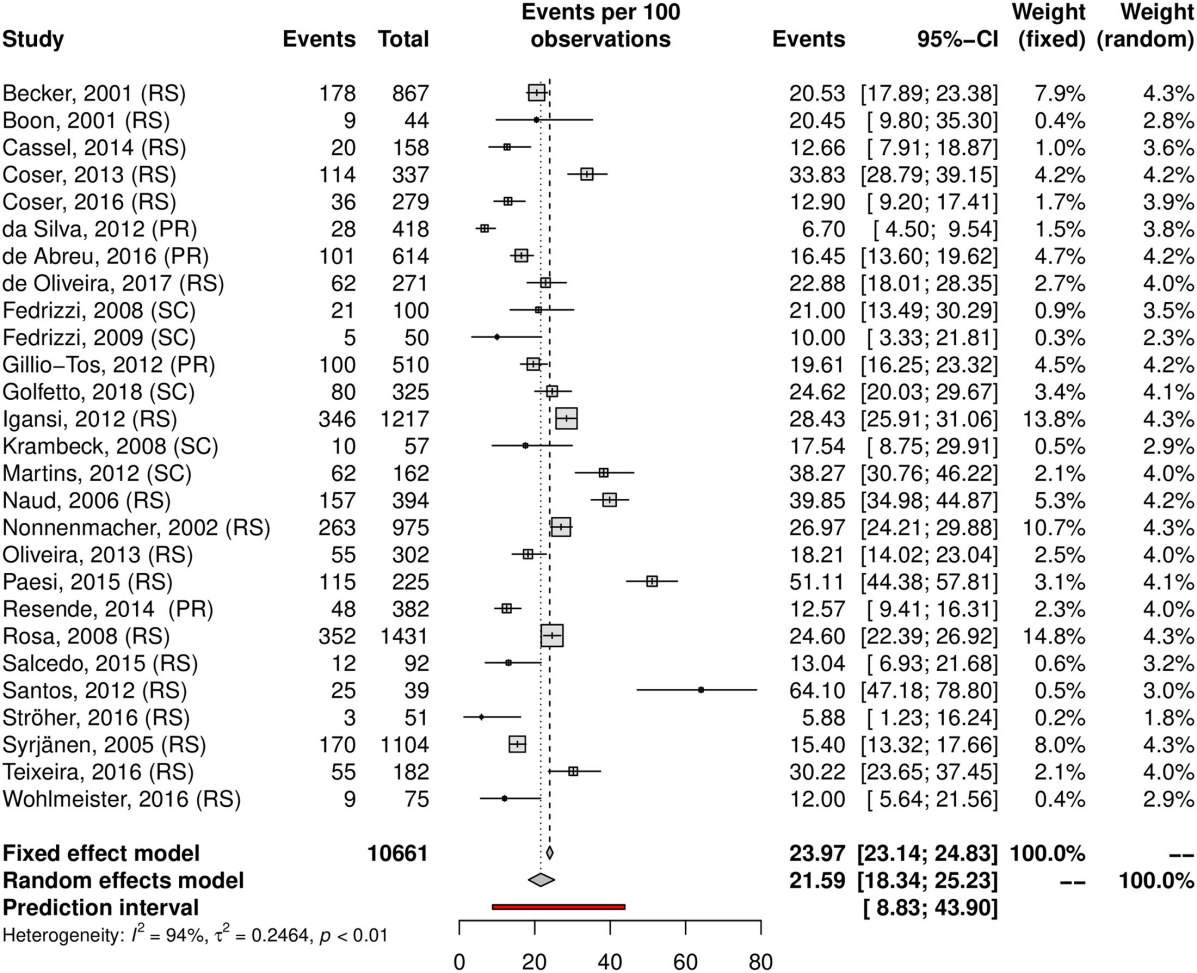
Prevalence of cervical HPV in the South region of Brazil. Forest plot of a metanalysis of studies reporting prevalence of cervical infection by HPV in the South region of Brazil.

**Fig 8:**
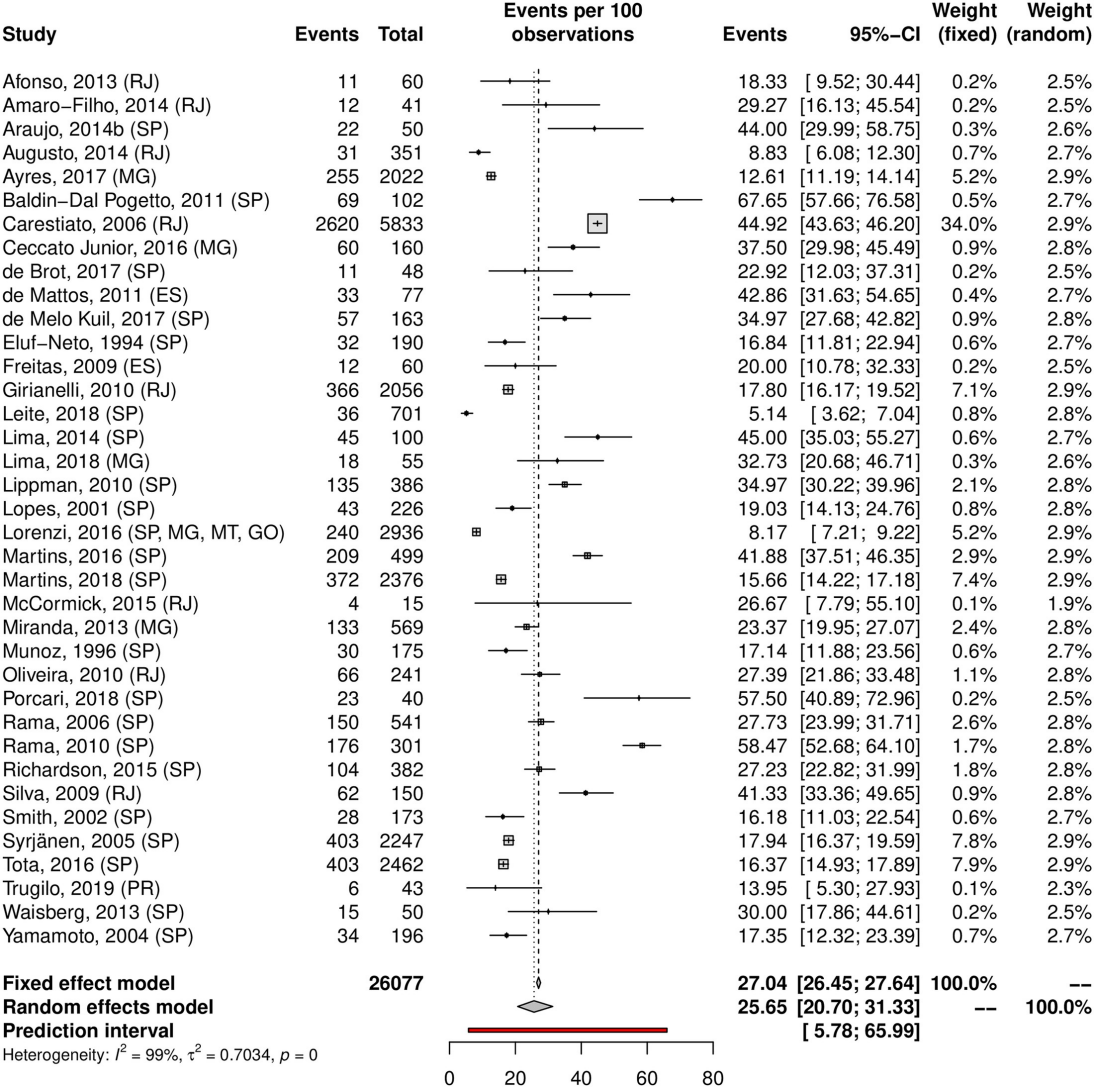
Prevalence of cervical HPV in the Southeast region of Brazil. Forest plot of a metanalysis of studies reporting prevalence of cervical infection by HPV in the Southeast region of Brazil.

**Fig 9:**
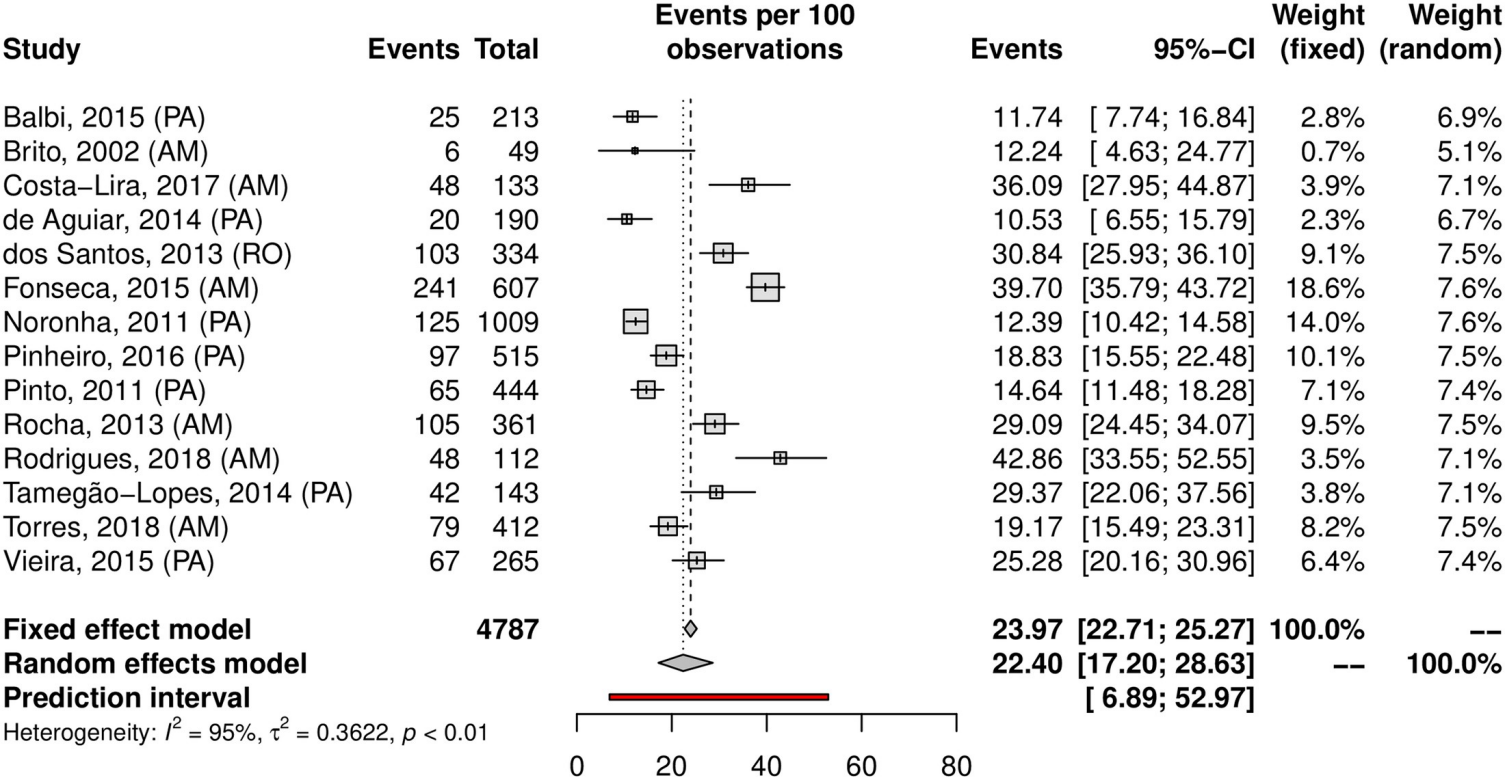
Prevalence of cervical HPV in the North region of Brazil. Forest plot of a metanalysis of studies reporting prevalence of cervical infection by HPV in the North region of Brazil.

**Fig 10:**
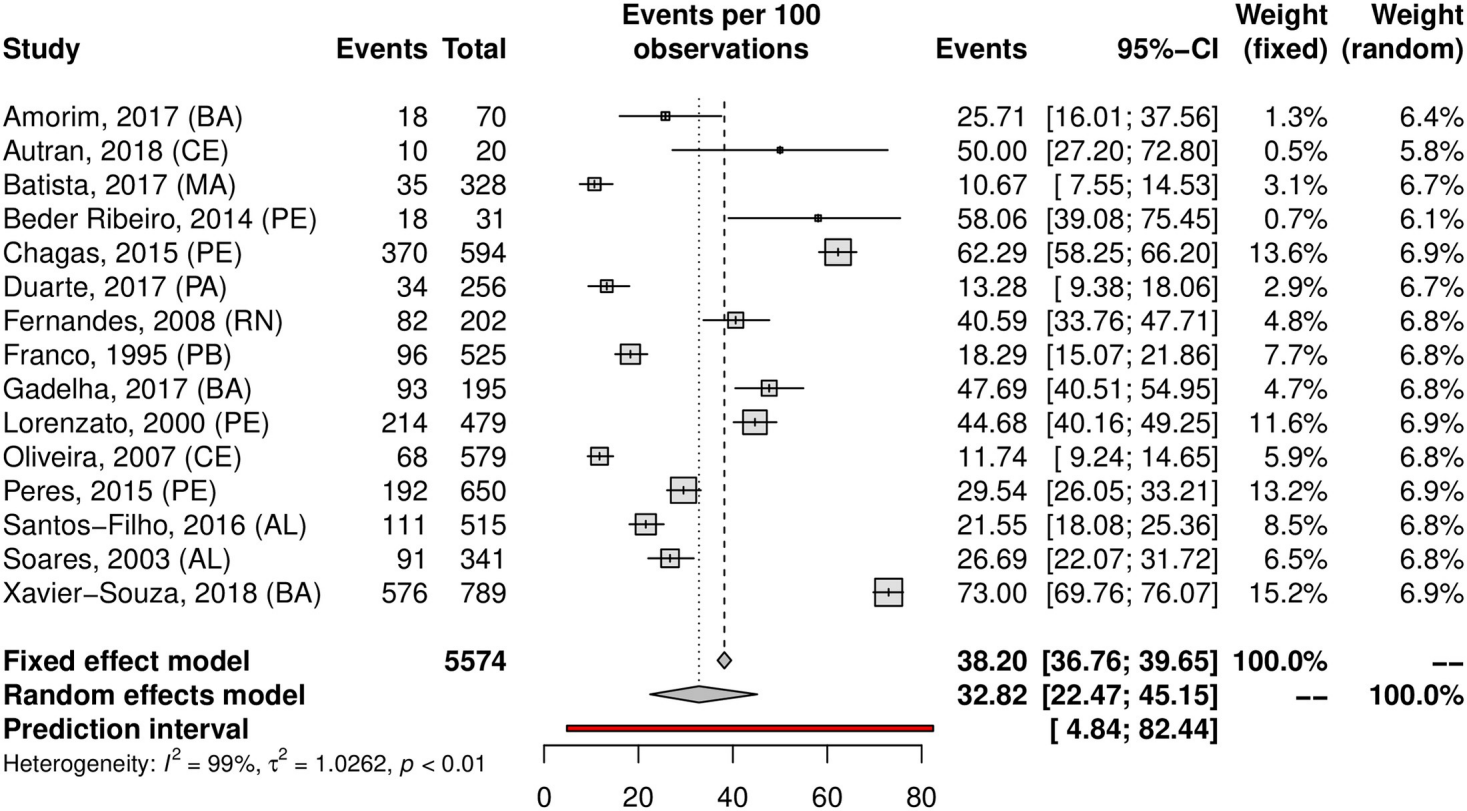
Prevalence of cervical HPV in the Northeast region of Brazil. Forest plot of a metanalysis of studies reporting prevalence of cervical infection by HPV in the Northeast region of Brazil.

**Fig 11:**
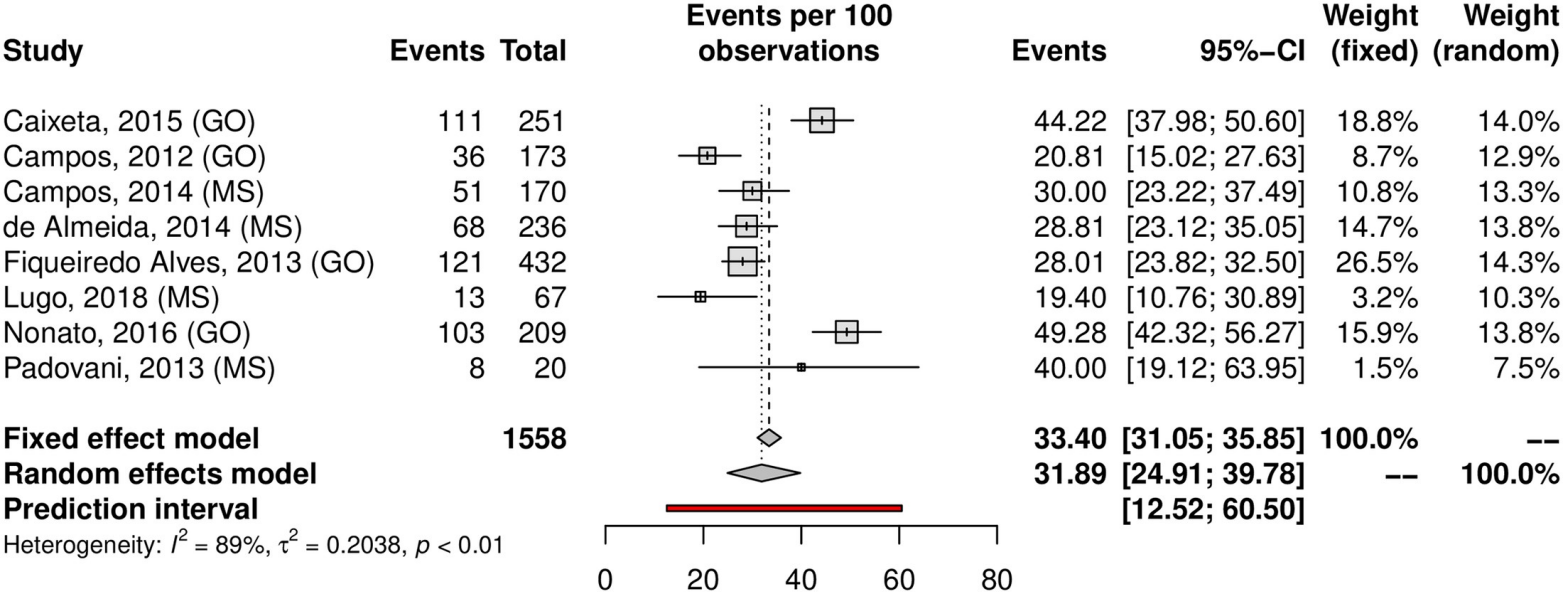
Prevalence of cervical HPV in the Central-West region of Brazil. Forest plot of a metanlysis of studies reporting prevalence of cervical infection by HPV in the Central-west region of Brazil.

A subgroup analysis was performed in an attempt to explain the heterogeneity among the studies. The HPV prevalence was 24.11% (95% CI 21.50–26.93; I^2^ = 98%) in low-risk populations (Fig 5) and 38.01% (95% CI 25.90–51.82; I^2^ = 97%) in high-risk populations (Fig 6). Most studies were carried out in the Southeast region (n = 37), followed by the South (n = 27) and Northeast (n = 15) regions; the North region was represented by 4 studies, while the Central-west region was represented by eight of them. Interestingly, HPV prevalence was slightly higher in the Northeast and Central-West areas (Table 1). The forest plots with the estimates for each region are shown in Figs 7–11. The prevalence of HR-HPV genotypes could also be analysed by region. Results show generally wider confidence intervals and a markedly lower prevalence in the South region, when compared to the other regions of Brazil (S3 Fig).

### Prevalence of HPV in penile samples

**Fig 12:**
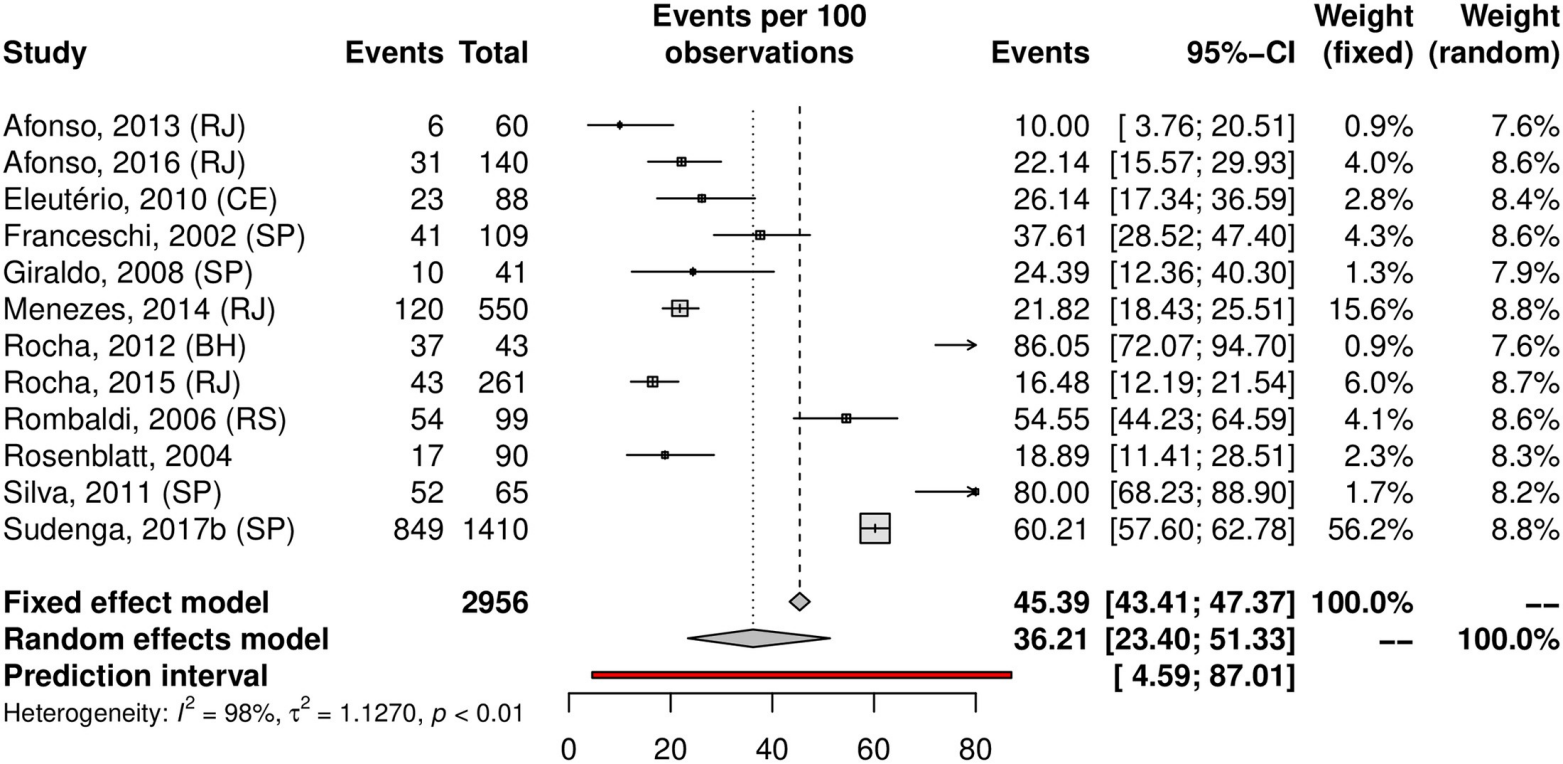
Overall prevalence of penile HPV. Forest plot of metanalysis of studies reporting prevalence of penile HPV infection in Brazil.

**Fig 13:**
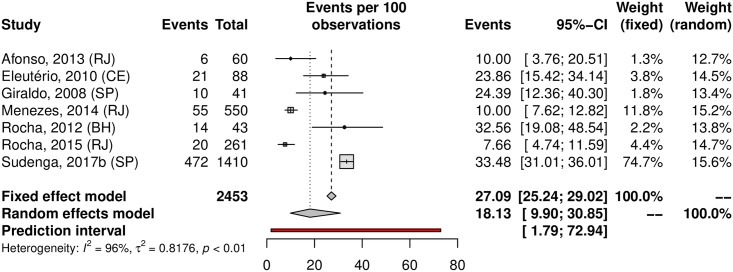
Overall prevalence of penile infection by high-risk HPV genotypes. Forest plot of a metanalysis of studies reporting prevalence of penile infection by HR-HPV genotypes in Brazil.

**Fig 14:**
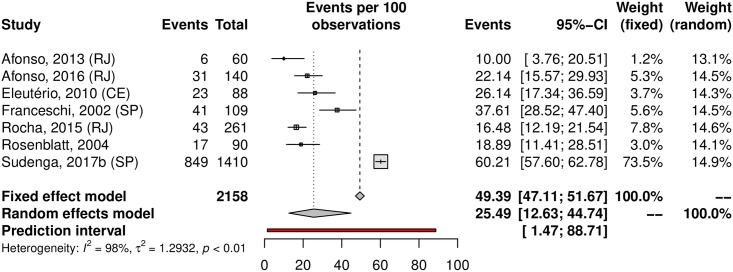
Prevalence of penile HPV infection in low-risk populations. Forest plot of a metanalysis of studies reporting prevalence of penile infection by HPV in low-risk populations in Brazil.

**Fig 15:**
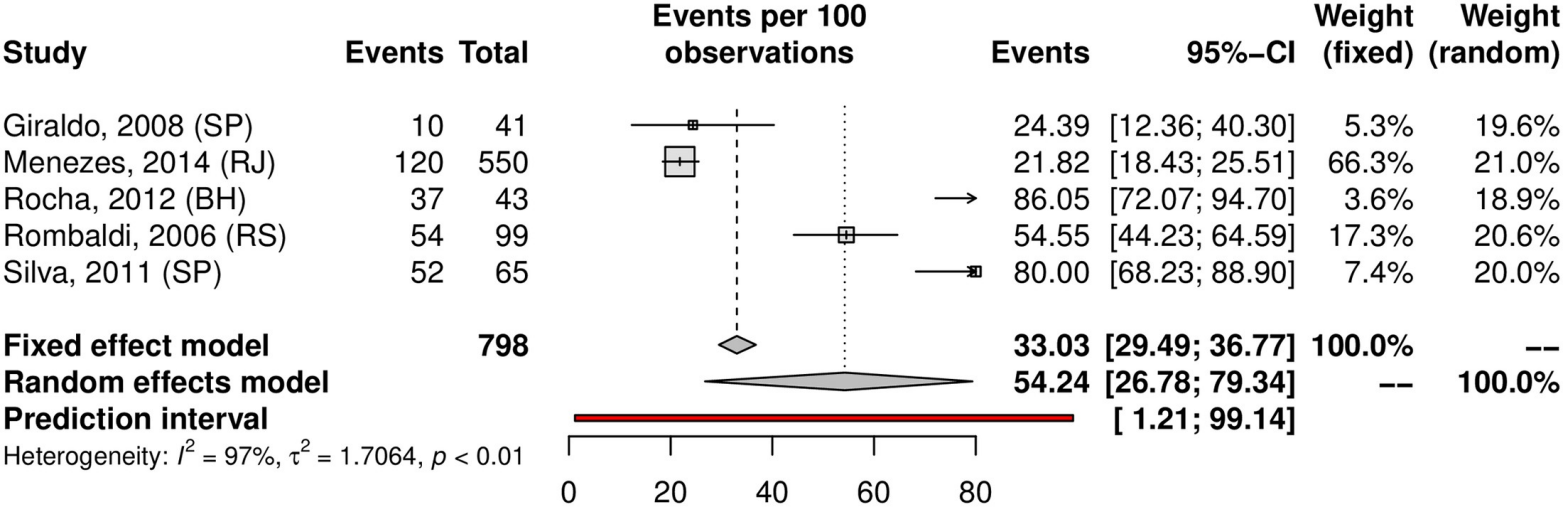
Prevalence of penile HPV infection in high-risk populations. Forest plot of a metanalysis of studies reporting prevalence of penile infection by HPV in high-risk populations in Brazil.

The prevalence of penile HPV was 36.21% (95% CI 23.40–51.33; 12 studies; I^2^ = 98%; Fig 12). The prediction interval for HPV prevalence ranged from 4.59 to 87.01%, with 95% confidence. Eight were conducted in the Southeast, with a prevalence of 39.61% (95% CI 22.54–59.64). Upon analyzing HR-HPV types, the studies reported a prevalence of 18.13% (95% CI 9.90–30.85; 7 studies; I^2^ = 96%; Fig 13). HPV prevalence in the low-risk population was 25.49% (95% CI 12.63–44.74; 7 studies; I^2^ = 98%; Fig 14). In the high-risk population was 54.24% (26.78–79.34; 5 studies; I^2^ = 97%; Fig 15).

### Prevalence of HPV in anal samples

**Fig 16:**
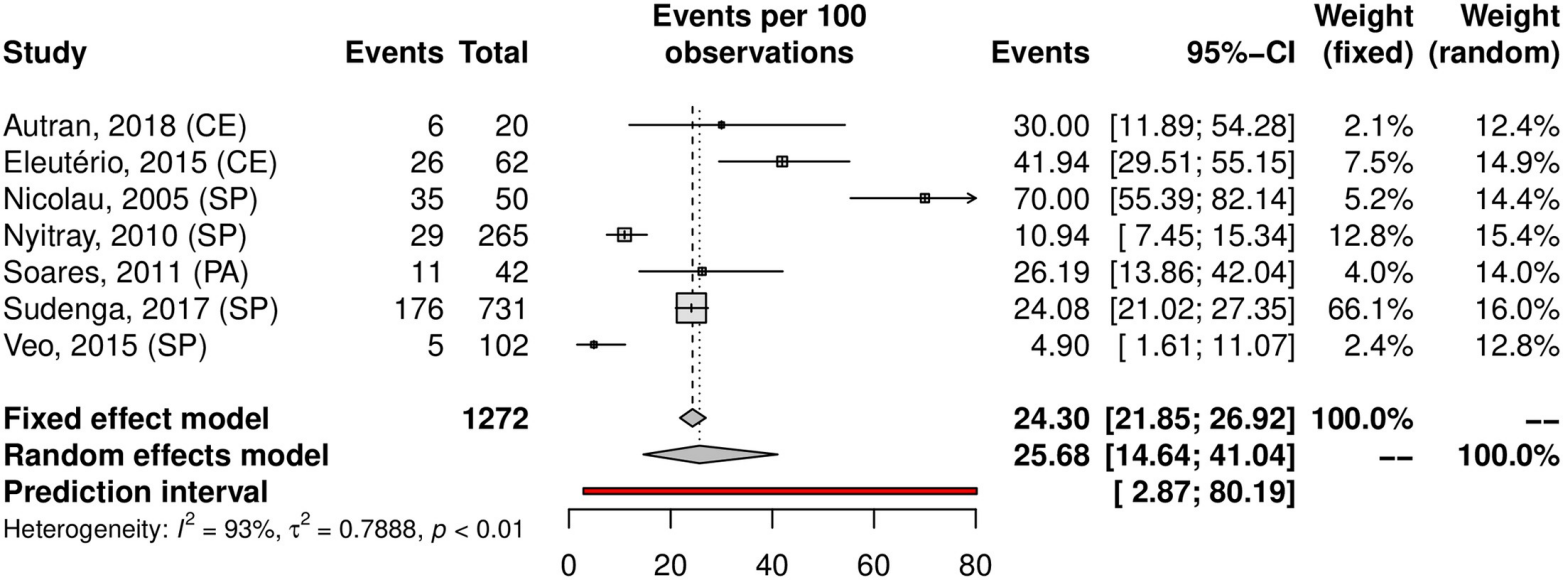
Overall prevalence of anal HPV infection. Forest plot of a metanalysis of studies reporting prevalence of anal infection by HPV in Brazil.

**Fig 17:**
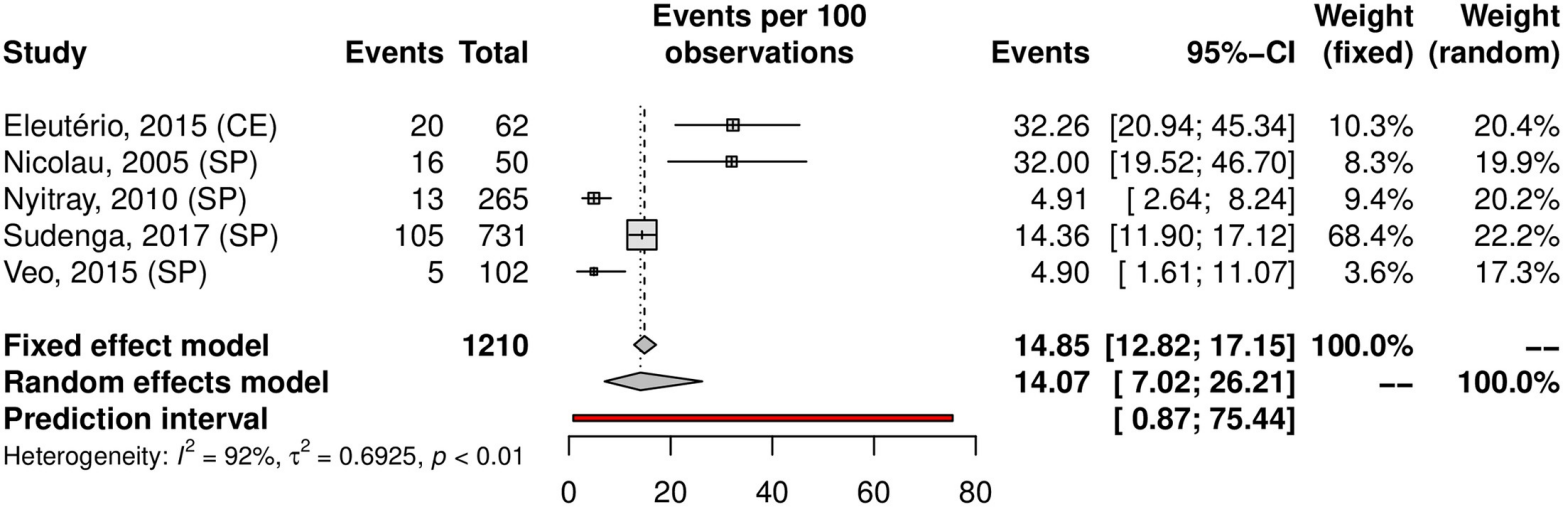
Overall prevalence of anal infection by high-risk HPV genotypes. Forest plot of a metanalysis of studies reporting prevalence of anal infection by HR-HPV in Brazil.

**Fig 18:**
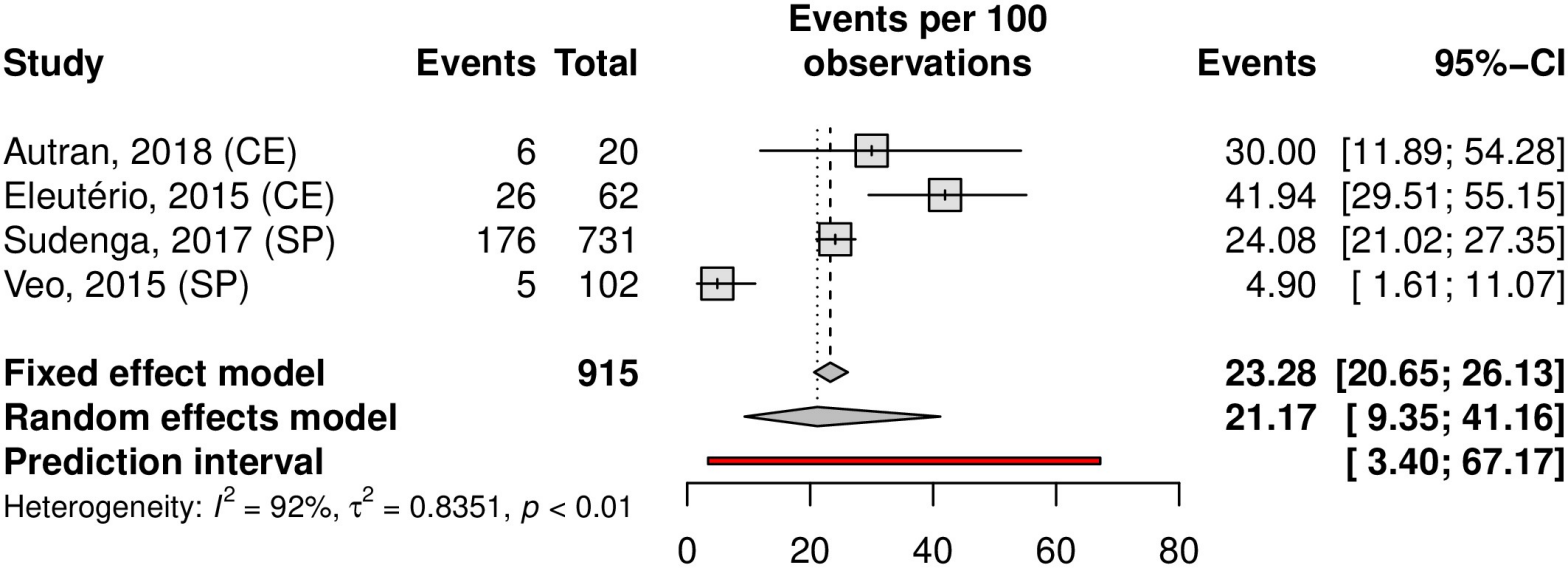
Prevalence of anal HPV infection in low-risk populations. Forest plot of a metanalysis of studies reporting prevalence of anal infection by HPV in low-risk populations in Brazil.

**Fig 19:**
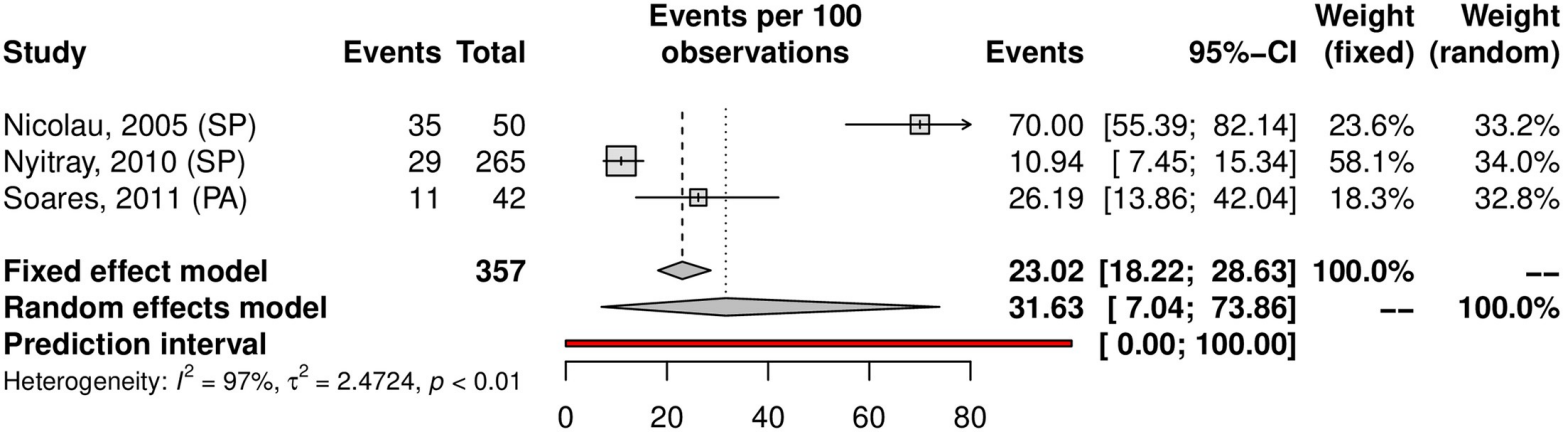
Prevalence of anal HPV infection in high-risk populations. Forest plot of a metanalysis of studies reporting prevalence of anal infection by HPV in high-risk populations in Brazil.

**Fig 20:**
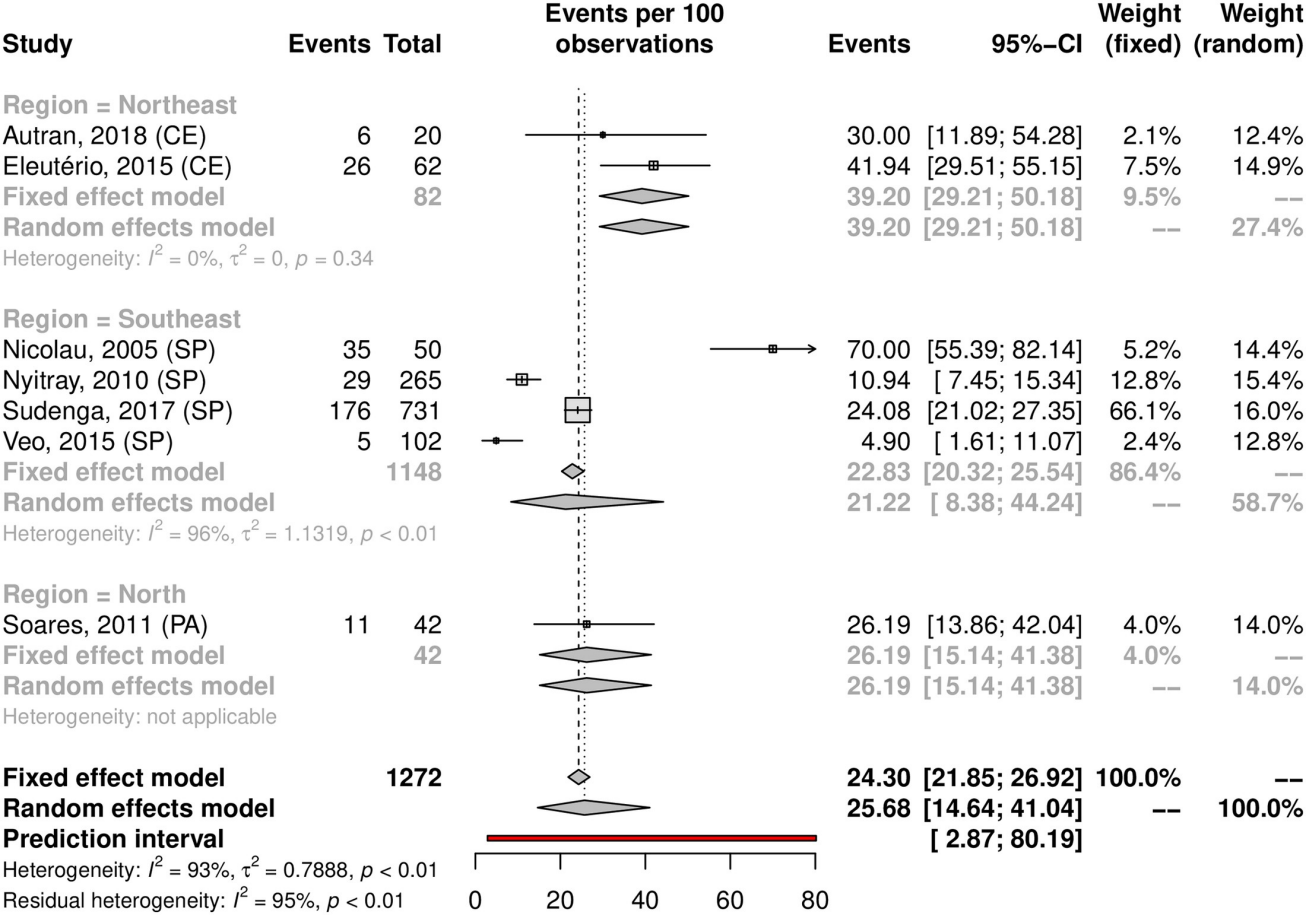
Prevalence of anal HPV infection in Brazil, by region. Forest plot of a metanalysis of studies reporting prevalence of anal infection by HPV, stratified by region in which each of them took place.

Among the analyzed studies, the prevalence of anal HPV was 25.68% (95% CI 14.64–41.04; 7 studies; I^2^ = 93%) as shown in Fig 16. The prediction interval for HPV prevalence ranged from 2.87 to 80.19%, with 95% confidence. The prevalence of HR-HPV types was 14.07% (95% CI 7.02–26.2). Eleuterio et al.[124] and Nicolau et al.[156] reported a prevalence of 32% compared to the 14% and 5% reported in three other studies [125,127,128] (Fig 17). This discrepancy might be explained by the HPV genotyping method (56.18% prevalence based on hybrid capture and 14.80% prevalence based on PCR). Evaluating sexual partners of women who had cervical HPV infection, Nicolau et al. a higher prevalence than in other studies was found (70%; 95% CI 55.39–82.14) [156]. The prevalence in the low-risk population was 22.10% (95%CI, 11.36–38.59; 4 studies; I^2^ = 89% Fig 18) and in the high-risk population was 31.63% (95% CI, 7.04–73.86; 3 studies; I^2^ = 97%; Fig 19). The prevalence of HPV among the Southeast, North and Northeast regions was 21.22%, 26.19% and 39.20%, respectively (Fig 20). There was no data on the HPV prevalence in the anal region in South and Central-West regions (Table 1).

## Prevalence of HPV in oral samples

**Fig 21:**
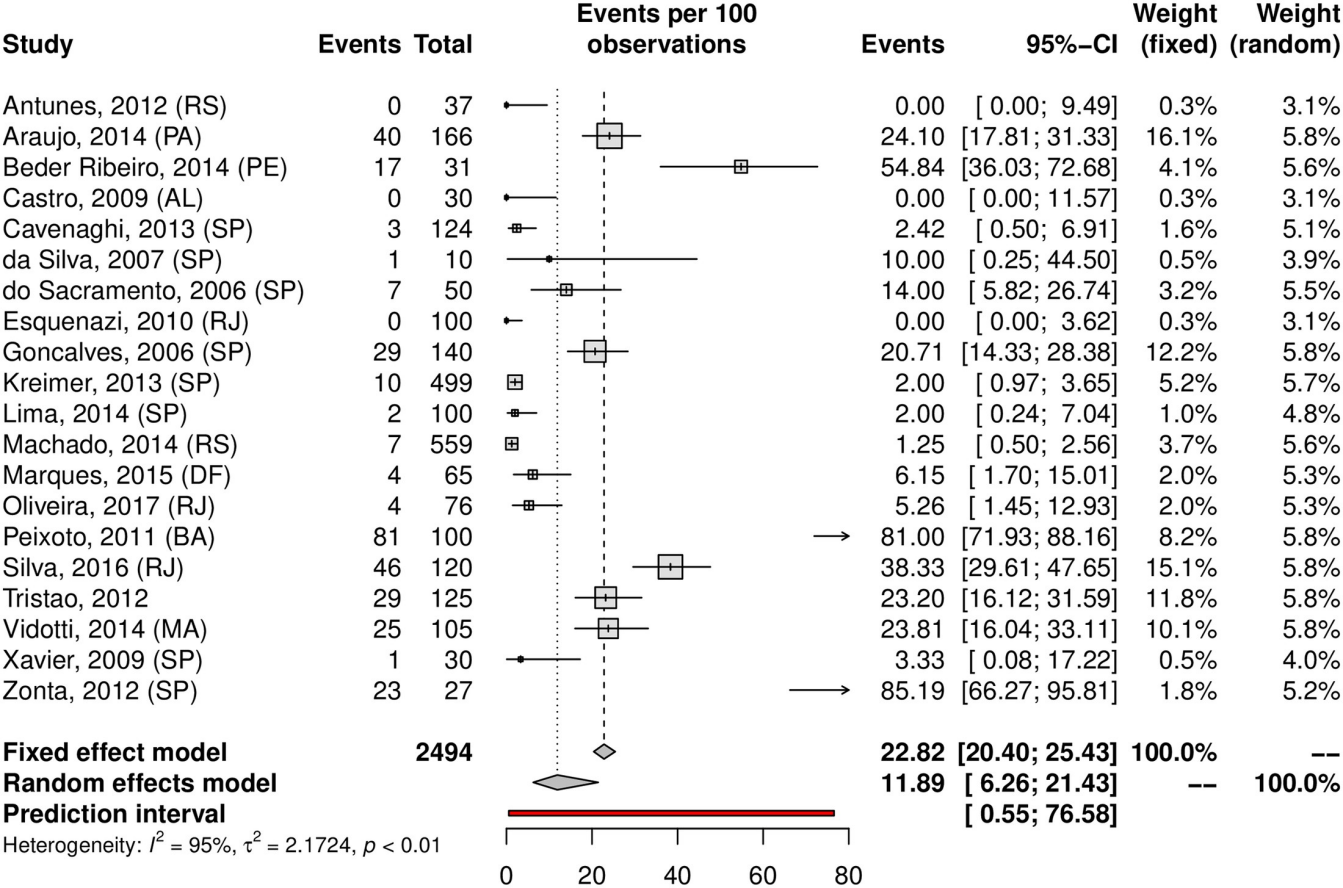
Overall prevalence of oral HPV infection. Forest plot of a metanalysis of studies reporting prevalence of oral infection by HPV in Brazil.

**Fig 22:**
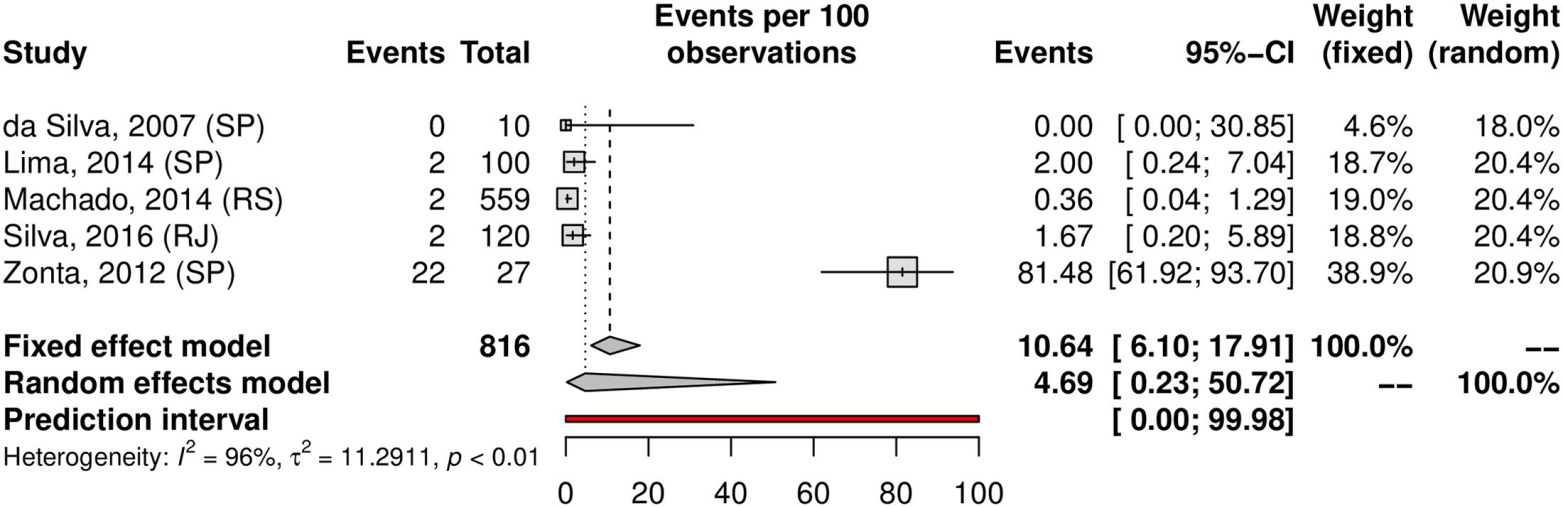
Overall prevalence of oral infection by high-risk HPV genotypes. Forest plot of a metanalysis of studies reporting prevalence of oral infection by HR-HPV in Brazil.

**Fig 23:**
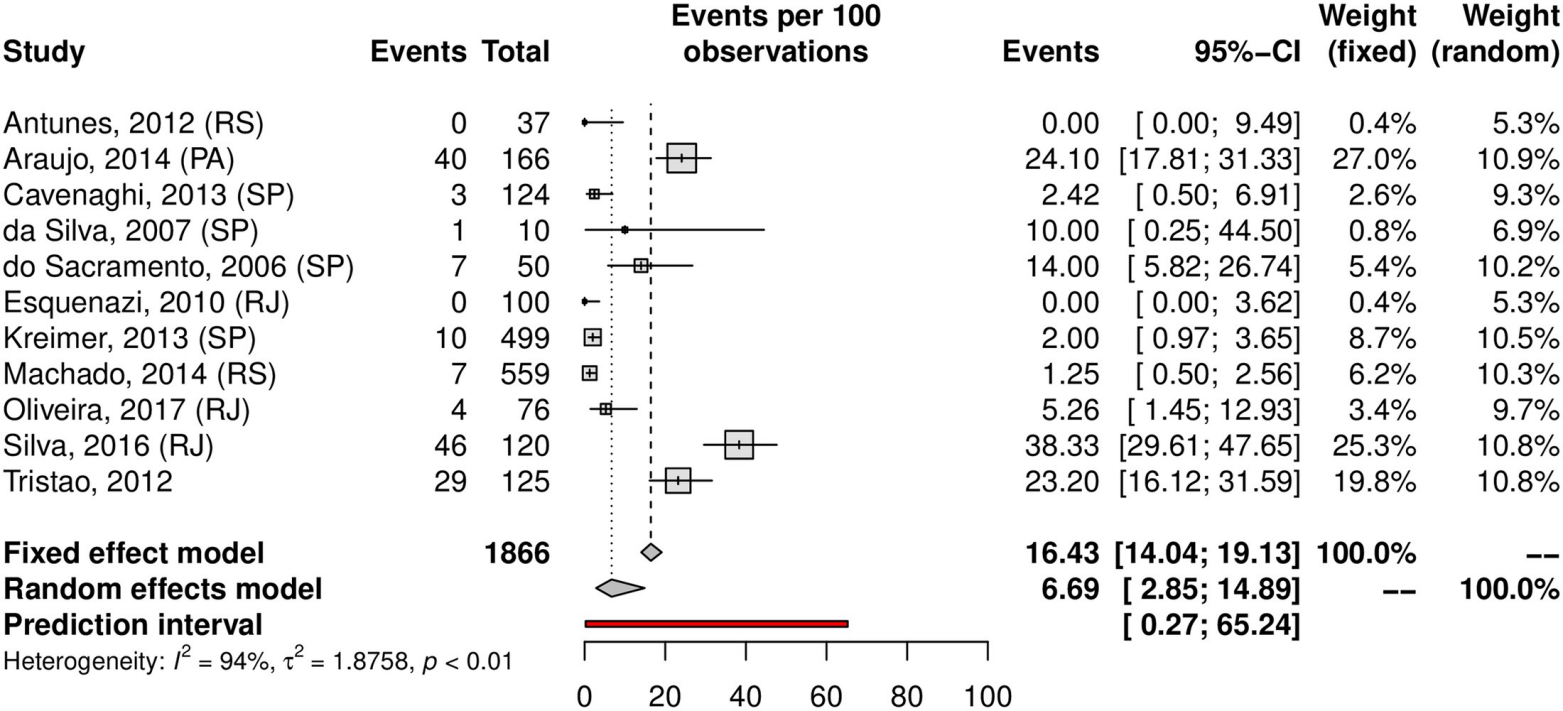
Prevalence of oral HPV infection in low-risk populations. Forest plot of a metanalysis of studies reporting prevalence of oral HPV infection in low-risk populations in Brazil.

**Fig 24:**
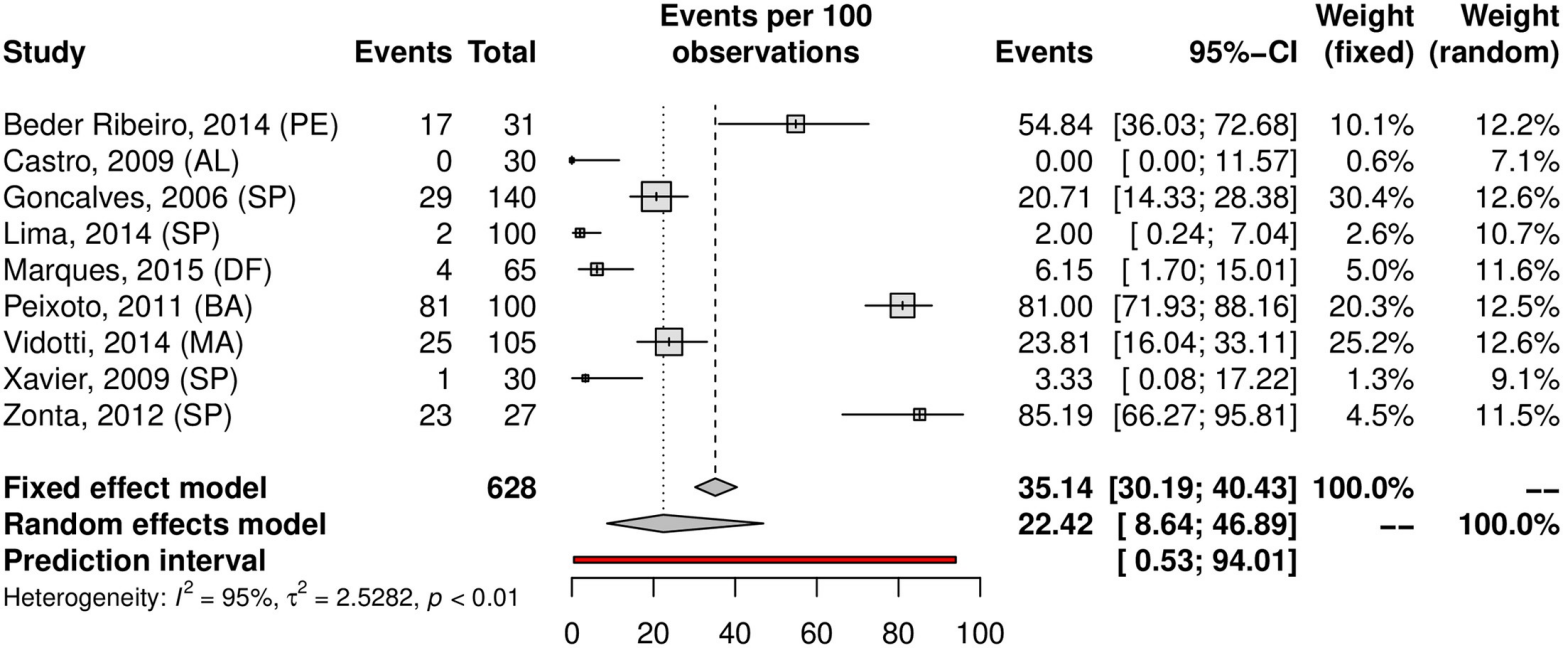
Prevalence of oral HPV infection in high-risk populations. Forest plot of a metanalysis of studies reporting prevalence of oral HPV infection in high-risk populations in Brazil.

**Fig 25:**
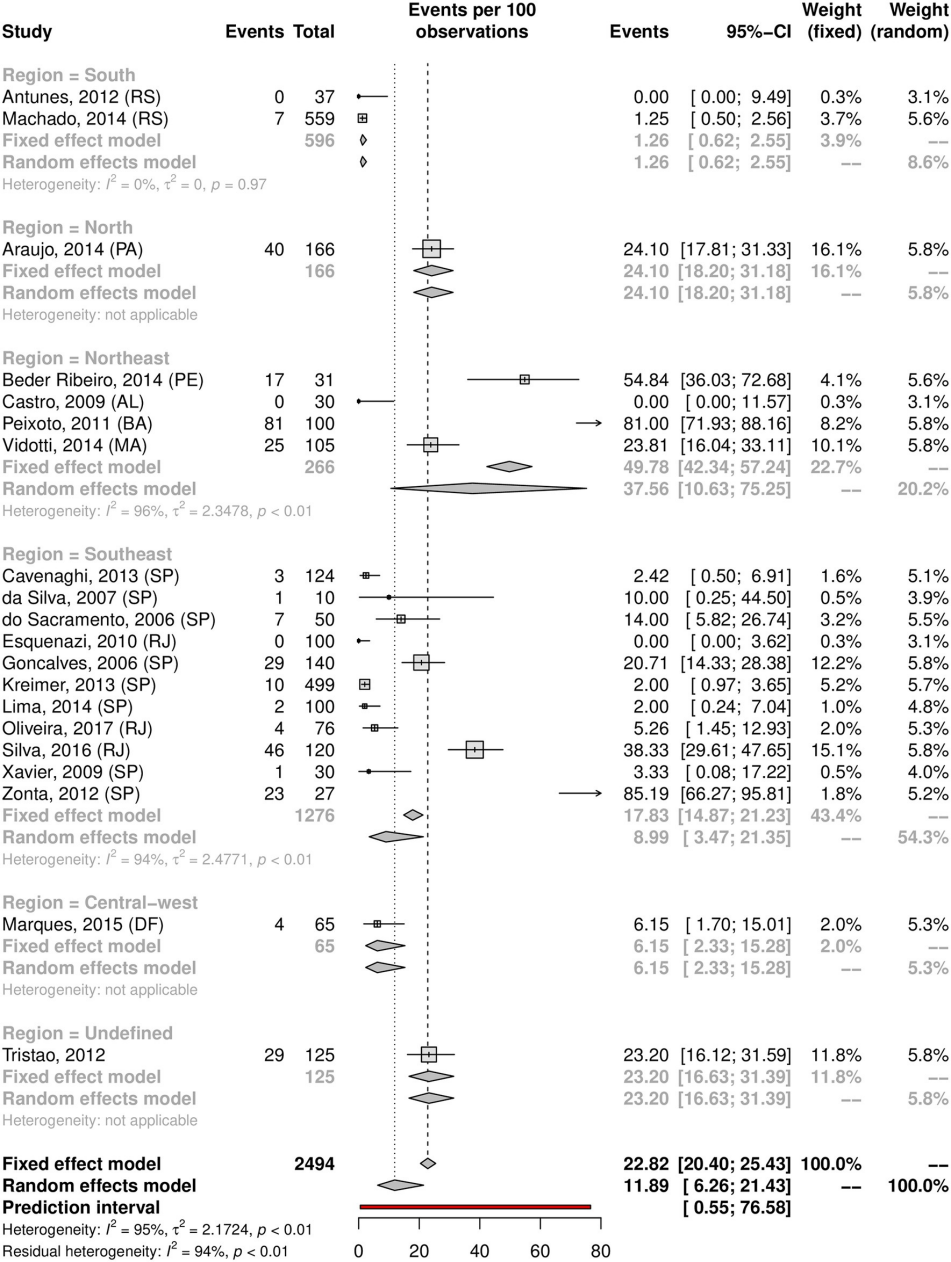
Prevalence of oral HPV infection in Brazil, by region. Forest plot of a metanalysis of studies reporting prevalence of oral infection by HPV, stratified by region in which each of them took place.

HPV prevalence in the oral region was 11.89% (95% CI 6.26–21.43; 20 studies; I^2^ = 95%; prediction interval 0.55 to 76.58%) (Fig 21), and the prevalence of HR-HPV types was 4.69% (95% CI 0.23–50.72; 5 studies; I^2^ = 96%; Fig 22). The prediction interval for HPV prevalence ranged from 0.55 to 76.58%, with 95% confidence. The subgroup analysis showed an HPV prevalence of 6.69% (95% CI 2.85–14.89; 11 studies; I^2^ = 94%; Fig 23) in the low-risk group and 22.42% (95% CI 8.64–49.89; 9 studies; I^2^ = 95%; Fig 24) in the high-risk group. The majority of studies were published in women and conducted in the Southeast followed by the Northeast and South regions. There was an important difference in oral HPV prevalence between studies (from 0% to 85.19%), with overall results being higher in the Northeast (37.56%) compared to that of Central-West and South regions (6.15% and 1.26%, respectively); the North and Central-West regions each had only one study included (Table 1) (Fig 25).

## Discussion

This systematic review included more than 50,000 people and showed that Brazilian population have a high prevalence of HPV infection as compared to women with normal cytology in different world regions such as Central America (13%), Northern Africa (9.2%), Western Europe (9%) and Southern Asia (7.1%) [3,158]. The highest prevalence was found in the penile region with 36% followed by cervical and anal region with approximately 25% and then oral region with 12%. There is a variation in the HPV prevalence among different geographic areas in Brazil, with increased prevalence in the Northeast (except at the penile site) and scarce data from the North and Central-West geographic regions. To the best of our knowledge, this is the first study that has evaluated the prevalence of HPV at multiple sites on the body and different Brazilian regions in general population.

Overall prevalence of HPV and the prevalence of high-risk HPV differs slightly across regions in Brazil. Most studies included in this review were carried out in the South and Southeast regions. This was an expected result, as these are highly populated and the most economically developed regions of Brazil [159]. Cervical HPV prevalence was higher in the Central-west and Northeast regions. Few of the studies retrieved in our review were from these regions, and this could have an influence on the estimate. Alternatively, it could be that these differences are real, and they could be related to socioeconomical aspects, in particular when this analysis is focused in the Northeast region, which is highly populated, poorer and has worse general health indicators [159,160].

Our study showed a prevalence of cervical HPV of 24.11% and 38.01% in low- and high-risk populations, respectively. According to a meta-analysis of one million women, the estimated global HPV prevalence in women with normal cytological was 11.7%, but it was 16.1% in Latin America [3]. However, similar to our results, a previous review published in Brazil reported a cervical prevalence ranging from 13 to 54%, with most of the data representing the Southeast population [5]. With this systematic review, we focused on existing data regarding HPV prevalence and expanded the overall summaries to a national scope. The regional-specific HPV prevalence varies from 20–35%. It is important to highlight that the North region, which has the highest incidence of cervical cancer in Brazil (25.62/100.000) [9], did not present a higher prevalence of HPV according to our results. It could be the case that studies from the North region underestimated HPV prevalence, or, conversely, that prevalences in other regions are overestimated. Another reason for this apparent contradiction is less accessibility to cervical cancer screening programs and less access to health care in the North region of Brasil [161].

It was not possible to pool data for specific genotypes beyond HPV-16 and HPV-18. Our results show a relatively high prevalence HPV-18 in females when compared to data from the USA [162] and other countries [163]. While we attempted to address selection bias by categorizing high-risk and low-risk populations, it was not possible to completely eliminate it. Patients who sought screening programs or medical care could have different risk profiles than those who did not.

The HPV prevalence in the penile region was 25.49% in the people without clinical lesions and even higher among high-risk populations (54.24%). According to a previous systematic review, genital HPV infection in men ranges from 1.3% to 72.9% [164], and the HIM study reported an HPV prevalence of 72% among Brazilian men [165]. The reason for this high prevalence might be due to sampling differences among the studies, which includes the scrotum and/or penis, a larger sample size and the inclusion of a variety of recruitment strategies such as reference centers for urogenital care and general media advertising [165]. Furthermore, differences in the laboratory technique could partially explain the difference. The evaluation of penile HPV is underrepresented in most Brazilian regions, and the available studies have a large number of differences regarding their design. The Southeast and South regions tend to show higher HPV prevalence when compared to the Northeast, but the Southeast was the only region that published more than two studies. For example, one of the two Northeast studies represented a low-risk population [117], whereas the study originating from the South region included the partners of women with CIN [121].

The prevalence of HPV in the anal region was similar to that of the cervical region and comprised 25.68% of population. Anal HPV prevalence is typically higher among high-risk groups, which is in accordance with previous studies [4]. There were a limited number of studies in Brazil that evaluated HPV in this anatomic site, and four were conducted in Southeast. The available data on the general population are comparable with the prevalence of cervical HPV and could be explained by concomitant HPV infection in both anal and cervical regions.

Prevalence of cervical HPV in Brazil is higher than in other developing countries. A past review has estimated generally lower prevalence of HPV in the cervix of healthy females in most developing and developed countries, but some of this discrepancy could be explained by the broader inclusion criteria of our study [163]. While the prevalence of male genital HPV varies substantially among developing countries, most of them reported prevalences around 20%, which is higher than in developed countries but lower than our findings [166]. Finally, extending this comparison to oral HPV, our estimates seem to be in accordance to those found for South American countries in another review. That study also found that South America was the continent with the highest prevalence of HPV, at 12.4%, significantly above both Asia, at 2.6% and Africa, at 7.0%, and also higher than Oceania (4.6%), North America (7.7%) and Europe (9.9%) [167].

This meta-analysis shows an oral HPV prevalence of 6.69% among the population without clinical lesions and 22.42% within the high-risk population. As is commonly known, oropharyngeal cancers are associated with tobacco and alcohol intake, but the incidence of HPV-induced cancers increased and HPV infection is now recognized as an important etiological factor in this disease [168,169]. A previous systematic review developed in Brazil showed a 6% HPV prevalence among the healthy population but a higher prevalence (38.5%) within high-risk groups [170]. Although that review was developed using a different data analysis model compared to our model, the results of both studies were highly comparable. Similar to the other analysis of oral HPV prevalence, most of the studies were conducted in the Southeast region. The higher prevalence in the Northeast might be attributed to the inclusion of the high-risk population within the analyses. Although most of the studies included men and women, the reported data were insufficiently stratified to allow for analysis by gender. In addition, the studies that only included women generally evaluated participants with concomitant genital HPV. There was a high heterogeneity in HPV oral prevalence among the studies in Brazil, ranging from zero to 96%. The two studies with the highest oral HPV prevalence, around 80%, investigated the presence of oral HPV DNA in women with diagnosis of genital HPV [141,146].

In terms of public health policy, our results suggest that while some efforts could be targeted towards regions and populations with higher HPV prevalence, broad approaches [171] seem appropriate, as illustrated by the fact that the prevalence of HPV is high even in low-risk populations and that it differs only slightly across regions. Additionally, the high prevalence of HPV in anatomic sites other than the cervix suggests it should not be approached as a problem restricted to cervical cancer.

This study is a comprehensive systematic review that used a broad search strategy, including large studies that analyzed different anatomic sites and represented different geographic regions throughout the country. Additionally, the findings of this systematic review increase our understanding of overall and HR-HPV prevalence within the general population in Brazil and by geographic region. We understand that the included studies did not accurately represent a sampling of Brazilian cities; however, this review provides the best estimated data on HPV prevalence to date.

Our study has some limitations. Brazil is a vast country, and the prevalence of HPV in some regions was either reported in a small number of studies or not reported at all. Thus, the population included in our review may not be representative of the general population throughout Brazil. The high heterogeneity among studies may impact in the exact prevalence measured. In contrast with randomized trials, non-controlled studies, such as the included in this systematic review, have smaller sample size and consequently higher imprecision and statistical inconsistency. Given that the estimates of individual studies are included in the prediction interval, we hypothesize that most of the heterogeneity are result of the different settings (for example, patients characteristics and age). Furthermore, participants of all ages were included in the present study, and it is known that HPV genital infection decrease with increasing age [172]. On this way, some discrepancies in prevalence could be due to differences in age range of included studies. As the majority of studies reported age in ranges, we could not aggregate the studies in age categories or perform meta-regression analysis. Most of the included studies looked at small groups and did not report the sample size statistical power; given the rarity of HPV infection at some anatomic sites (i.e., oral), a larger population-based sample size is needed to better evaluate HPV prevalence.

Prevalence studies are often used to devise public health actions and improve health services as well as serve as the first step to evaluate national control programs. The overall quality of the evidence for HPV prevalence was low or very low for all anatomic sites. In 2014, Brazil launched a national HPV vaccination program that includes girls between 9 and 13 years old and since 2017 the program started including boys of 12 and 13 years of age. Robust evidence is important for adequate monitoring of the populational impact of the program across Brazil. In view of the lack of proper national evidence regarding the HPV prevalence and databases tracking HPV infection rates, as well as to meet the needs of the Brazilian Ministry of Health, we conclude that is necessary to develop a nationwide study to investigate HPV presence among the Brazilian population. A nationwide HPV prevalence study (POP-Brazil study) with young adults between 16–25 years is in development, and it is predicted that the results of this study will fulfill the lack of epidemiological information detected in this review [173].

In summary, the prevalence of HPV in Brazilian population is high and varies by anatomical body site, with lower rates in the oral cavity compared to that in the cervical, penile and anal region. Studies on HPV have been primarily developed to evaluate infection and cancer in the cervical region. There is a profound lack of HPV data in many geographic regions of Brazil and for different anatomic sites.

## Supporting information

S1 PRISMA checklist PRISMA 2009 checklist. (PDF)

S1 Appendix Details of electronic bibliographic database search strategies. (PDF)

S1 Table Characteristics of studies included in the systematic review. (PDF)

S2 Table Assessment of bias risk. (PDF)

S1 Fig Funnel plot of the studies that measured the prevalence of (a) cervical, (b) penile, (c) anal and (d) oral region. (PDF)

S2 Fig Overall prevalence of cervical HPV in Brazil. (PDF)

S3 Fig Sub-group analysis of prevalence high-risk HPV genotypes in Brazil, by region. (PDF)
