# Supplementary material for: Prevalence of human papillomavirus (HPV) in Brazil: A systematic review and meta-analysis
Source: PLoS One. 2020 Feb 21;15(2):e0229154. doi: 10.1371/journal.pone.0229154 (PMC7034815; doi:10.1371/journal.pone.0229154)
Supplement: S1 Appendix — (PDF) [file pone.0229154.s002.pdf]

Additional File 1. Details of electronic bibliographic database search strategies

Medline (via Pubmed)

(((((Brasil*) OR Brazil*)) OR Brazil[mesh]

AND

((("human papillomavirus") OR ("Human papillomavirus 31"[Mesh] OR "Human papillomavirus 6"[Mesh] OR "Human papillomavirus 16"[Mesh] OR "Human papillomavirus 18"[Mesh] OR "Human papillomavirus 11"[Mesh])) OR HPV)))

AND

((human) NOT animal)

Embase

'human papillomavirus type 11'/exp OR 'human papillomavirus type 16'/exp OR 'human papillomavirus type 18'/exp OR 'human papillomavirus type 31'/exp OR 'human papillomavirus type 33'/exp OR 'human papillomavirus type 35'/exp OR 'human papillomavirus type 45'/exp OR 'human papillomavirus type 52'/exp OR 'human papillomavirus type 58'/exp OR 'human papillomavirus type 59'/exp OR 'human papillomavirus type 6'/exp OR 'human papillomavirus type 73'/exp OR 'Wart virus'/exp OR 'human papillomavirus':ab,ti OR 'hpv':ab,ti

AND

('brazil'/exp OR brazil*:ab,ti OR brasil*:ab,ti)

NOT

[animals]/lim

Lilacs

(HPV OR Human papillomavirus OR Papillomavirus humano)

AND

(Brasil$ OR Brazil$)

Scielo

("human papillomavirus" OR "papillomavirus humano" OR "human papilloma virus" OR HPV)

AND

(Brasil* OR Brazil*)

ISI - Web of Knowledge

TS= ("human papillomavirus" OR HPV)

AND

TS=(Brazil* OR Brasil*)
