# Supplementary material for: Prevalence of human papillomavirus (HPV) in Brazil: A systematic review and meta-analysis
Source: PLoS One. 2020 Feb 21;15(2):e0229154. doi: 10.1371/journal.pone.0229154 (PMC7034815; doi:10.1371/journal.pone.0229154)
Supplement: S1 Table — (PDF) [file pone.0229154.s003.pdf]

Author, year	Region, State	Study design	Population	Proportion of Women (%)	N	Age (mean)	Age (range)
**Cervical region**							
Alvarez-Arguelles, 2009	Undefined	Abstract Meeting	Women attending Sexual infection disease control program	100	472	28.9	18-75
Amaro Filho, 2014	Southeast, RJ	Cross-sectional	Women selected from the archives	100	87	51.1	24-88
Amorim, 2017	Northeast	Cross-sectional (case-control)	Women seeking medical consultations, with a positive diagnosis for cervical lesions (Cervical Intraepithelial Neoplasia (CIN) 1, 2 and 3 or in situ carcinoma) were selected as the case group. The controls (women without cervical lesion) were from the Primary Care Units of Public Health Clinics (SUS – Brazil) also located in Vitória da Conquista city, Bahia, Brazil.	100	132	Control: 37.6 / Case: 38.7	14-78
Araujo, 2014b	Southeast, SP	Cross-sectional	Athletes	100	50	20	-
Augusto, 2014	Southeast, RJ	Cross-sectional	Women referred to the Family Health Program	100	351	38.4	14-79
Ayres, 2017	Southeast, MG	Cross-sectional	Women living in the public health care coverage area between 20 and 59 years of age, asymptomatic	100	2076	20–24: 258<br>25–29: 279<br>30–34: 303<br>35–39: 280<br>40–44: 274<br>45–49: 246<br>50–54: 222<br>55–59: 200	20-59
Balbi, 2015	North, PA	Thesis	Women attended at gynecology ambulatory	100	213	50.7	35-60
Baldin-Dal Pogetto, 2011	Southeast, SP	Cross-sectional	Female sex workers	100	102	26.1	-
Batista, 2017	Northeast, MA	Cross-sectional	Women 15 to 75 years of age from the municipalities of the east coast of Maranhão in the mentioned communities and living and registered in the areas covered by family health teams.	100	353	<30: 99<br>31-40: 88<br>41-50: 72<br>51-60: 59<br>>60: 35	-
Becker, 2001	South, RS	Cross-sectional	Women from Women`s Anti-Cancer Program	100	977	39.1	16-70

Boon, 2001	South, RS	Case-control	Female partners of HIV (+) infected men	100	44	28.5	-
Brito, 2002	North, AM	Cross-sectional	Indigenous women	100	49	25	10-73
Caixeta, 2015	Central-West, GO	Cross-sectional	Users of public health system	100	251	19.4	15-25
Campos, 2012	Central-West, GO	Case-control	Anticancer Association and Basic Health Units	100	173	-	16-48
Campos, 2014	Central-West, MS	Cross-sectional	Patients from Municipal Laboratory	100	170	NI	18-65
Cassel, 2014	South, RS	Cross-sectional	Normal cervical smears from healthcare medical center	100	158	42.7	-
Ceccato Junior, 2015	Southeast, MG	Cross-sectional	HIV (-)	100	160	35.3	-
Chagas, 2015	Northeast, PE	Cross-sectional	Women who volunteered for cervical cancer screening	100	594	-	-
Coser, 2013	South, RS	Cross-sectional	Women undergoing routine pap smear testing, public center	100	337	-	-
Coser, 2016	South, RS	Cross-sectional	Women who accepted to participate in the study while attending for cervical cancer screening in a primary public health care clinic	100	300	≤19: 28(9.3) 20-29: 56(18.7) 30-39: 59(19.7) 40-49: 60(20) 50-59: 59(19.7) ≥60: 38(12.7)	-
Costa-Lira, 2017	North, AM	Cross-sectional	women who used public health services	100	180	16-20: 23 21-30: 53 31-40: 71 41-50: 32	-
da Silva, 2012	South, PR	Cross-sectional	Women from cervical screening campaign	100	418	42.8	-
de Abreu, 2016	South, PR	Cross-sectional	Women attending basic health units of the Public Health System for cervical cancer screening consultations and reference services for colposcopy (Zona Sul Clinic) from August 2012 to March 2013	100	838	40.03	18-68

de Aguiar, 2014	North, PA	Cross-sectional	Incarcerated women	100	190	30.5	18-60
de Almeida, 2014	Central-West, MS	Cross-sectional	Young sexually active students	100	236	23.3	-
De Brot, 2017	Southeast, SP	Cohort	Women of any age and with no previous history of HPV vaccination who were consecutively and prospectively identified between	100	260	38 (median)	17-85
de Mattos, 2011	Southeast, ES	Cross-sectional	HIV negative patients	100	77	NI	14-51
de Melo Kuil, 2017	Southeast, SP	Cross-sectional	Women aged 18–64 years who were referred to the Colposcopy Ambulatory of the Prevention Department at Barretos Cancer Hospital (Brazil) due to abnormal (atypical squamous cells of uncertain significance or worse) cervical cytology test results (Pap smear)	100	204	<25: 19<br>25-45: 83<br>46-64: 61	18-64
de Oliveira, 2017	South, RS	Cross-sectional	Women attending the Gynecological Service of the University Hospital of Federal University of Rio Grande (HU-FURG), a regional reference where the Papanicolaou test was performed.	100	355 (HIV: 23.6% - 84)	31	-
dos Santos, 2013	North, RO	Cross-sectional	Women from Women's Health center	100	334	-	-
Duarte, 2017	Northeast, PA	Cross-sectional	Women living in riverside communities from 7 municipalities in the state of Pará participated voluntarily in the present study	100	353	37	16-81
Eluf-Neto, 1994	Southeast, SP	Case-control	Control group, women without invasive cancer, from 7 hospitals	100	190	52	-
Fedrizzi, 2008	South, SC	Cross-sectional	Samples from public and private clinic or hospital	100	100	-	-
Fedrizzi, 2009	South, SC	Case-control	Control group, samples from normal endometrium from public and private clinic	100	50	-	-
Fernandes, 2008	Northeast, RN	Cross-sectional	Women enrolled in cancer screening program	100	202	32	15-64
Fiqueiredo Alves, 2013	Central-West, GO	Cross-sectional	Sexually active women	100	432	17.2	15-19
Fonseca, 2015	North, AM	Cross-sectional	Indigenous women	100	661	35.8	10-92
Franco, 1995	Northeast, PB	Cross-sectional	Random sample of participants in a cervical cancer screening program	100	525	41.2	-

Freitas, 2009	Southeast, ES	Cross-sectional (case-control)	Control group, non-pregnant women from low socioeconomic groups	100	60	25.7	-
Gadelha, 2017	Northeast, BA	Cross-sectional	Women who attended healthcare units	100	195	40.3	17-82
Gillio-Tos, 2012	South, PR	Cross-sectional (case-control)	Women recruited by centers for cervical screening and campaigns	100	789	32	15-47
Girianelli, 2010	Southeast, RJ	Cross-sectional	Participants from Family Health Program	100	2056	-	-
Golfetto, 2018	South, SC	Cross-sectional	Women were attended for cervical screening in public clinics in São Miguel do Oeste, SC, Brazil	100	325	37	14-79
Holanda, 2006	Northeast, CE	Cross-sectional	Sexually active women living in rural districts	100	878	-	15-69
Igansi, 2012	South, RS	Cross-sectional	Non-symptomatic women at Primare Care Unit	100	1217	-	-
Krambeck, 2008	South, SC	Cross-sectional	Women attending gynecological clinics	100	97	32.6	15-60
Leite, 2018	Southeast, SP	Cross-sectional	Healthy women, 18 years-old or older, who spontaneously visited the outpatient care for cervical cancer prevention in a private Sao Paulo's hospital	100	725	39,7	18-77
Lima, 2018	Southeast, MG	Cross-sectional	Women examined in a general gynecology clinic and in a colposcopy referral center	100	126	<35: 65<br>>=35: 61	-
Lippman, 2010	Southeast, SP	Cross-sectional	Women from primary care clinic	100	818	-	-
Lopes, 2001	Southeast, SP	Cross-sectional	Incarcerated women	100	262	32.4	-
Lorenzato, 2000	Northeast, PE	Case-control	Institute of Mother and Child Health and the Cancer Hospital	100	479	36.5	13-84
Lorenzi, 2016	Southeast, SP, MG, MT, GO	Cross-sectional	Women volunteers were enrolled in an opportunistic screening program that was regularly performed in the Barretos Cancer Hospital	100	3068	47 (median)	18-85
Lugo, 2018	Central-West, MS	Cross-sectional	Female sex workers working in public places (squares, parks, gardens, streets, avenues, etc.) and private places (saunas, nightclubs, and brothels)	100	79	26.7	18-42
Martins, 2012	South, SC	Cross-sectional	Gynecological examination, in a private clinic setting	100	162	-	-
Martins, 2016	Southeast, SP	Cross-sectional	Women undergoing routine screening and women refered with a previous abnormal Pap test in the year preceding study enrollment from São Paulo and 99 from Barretos	100	665	43	14-95

Martins, 2018	Southeast, SP	Cross-sectional	Women undergoing opportunistic routine cervical cancer screening at the Projeto Região Oeste, São Paulo, Brazil	100	2376	43	14-86
McCormick, 2015	Southeast, RJ	Case-control	Control group, cervical specimens with normal cytology and colposcopy	100	15	-	-
Miranda, 2013	Southeast, MG	Cross-sectional	Random sample of participants in health units	100	569	37.7	-
Munoz, 1996	Southeast, SP	Cross-sectional (case-control)	Control group, women from 5 general hospitals	100	175	52.7	-
Naud, 2006	South, RS	Cross-sectional	Healthy women among the general population	100	500	NI	15-25
Nonato, 2016	Central-West, GO	Cross-sectional	Women under the age of 25 years monitored by the Family Health Program	100	276	19.5	15-24
Nonnenmacher, 2002	South, RS	Cross-sectional	Women with no history of conization attending a cancer screening program	100	975	-	-
Noronha, 2011	North, PA	Cross-sectional	Spontaneously Gynecological examination	100	1009	36.6	30-45
Oliveira, 2007	Northeast, CE	Cross-sectional	Women, assisted by the Family Health Program	100	592	31.5	-
Oliveira, 2010	Southeast, RJ	Cross-sectional	Sexually active women, no prior history of cervical abnormalities, from public school	100	241	19.6	-
Oliveira, 2013	South, RS	Cross-sectional	Women attending Gynecology and Obstetrics Ambulatories and Health Primary Units	100	302	32.7	-
Padovani, 2013	Central-West, MS	Cross-sectional	Cervical sample from Cancer Prevention Center	100	49	-	-
Paesi, 2015	South, RS	Retrospective cross-sectional	Women aged 20–60 years who were referred to the private Medical Center of Pathology, Caxias do Sul, Brazil, by a gynecologist for assessment of a cervical condition	100	250	32.5	20-60
Peres, 2015	Northeast, PE	Cross-sectional	Women receiving regular gynecological surveillance from 3 familial health units	100	673	-	18-60
Pinheiro, 2016	North, PA	Cross-sectional	Women enrolled in the Cervical Cancer Prevention Program (Programa de Prevenção do Câncer de Colo Uterino - PCCU) developed in riparian communities of four municipalities located in different geographical regions of Pará	100	516	Itaituba A: 40<br>Itaituba B: 39<br>Limoeiro de Ajurú: 37<br>Acará: 37<br>Bragança: 37	13-82
Pinto, 2011	North, PA	Cross-sectional	Women with intact uterus or cervix	100	444	36.9*	-

Porcari, 2018	Southeast, SP	Case-control	Women receiving regular gynecological surveillance from 3 familial health units	100	80	HPV-positive: 34<br>HPV-negative: 36	-
Rama, 2006	Southeast, SP	Cross-sectional	Healthy, sexually active women looking for care	100	541	-	-
Rama, 2010	Southeast, SP	Cross-sectional	Primiparous women who gave birth after > 32 weeks of gestation	100	301	19.9	-
Resende, 2014	South, RS	Case-control	Control group, women without cytological abnormalities	100	382	-	-
Richardson, 2015	Southeast, SP	Cross-sectional (case-control)	Women attending for regular screening at 1 of 3 participating public hospitals	100	1058	-	-
Rocha, 2013	North, AM	Cross-sectional	Sexually active women using health services	100	364	36.4	18-78
Rodrigues, 2014	North	Cross-sectional	Sexually active women	100	84	31.1	10-66
Rodrigues, 2018	North, AM	Cross-sectional	Non-indigenous HIV-infected and HIV-uninfected women living in the Tapajós region	100	153	36,9	17-75
Rosa, 2008	South, RS	Cohort	Asymptomatic women attending a primary care clinic	100	1431	-	-
Roteli-Martins, 2011	Undefined	Cross-sectional	Healthy women with an intact uterus	100	3204	20.2	-
Salcedo, 2015	South, RS	Cross-sectional	Non-pregnant women, with no history of cervical cancer	100	183	31.9	-
Santos, 2012	South, RS	Cross-sectional	Self- collection cervicovaginal sample	100	39	-	-
Santos, 2016	Northeast, AL	Cross-sectional	Patients attending public and private health centers	100	515	15-25: 121<br>26-40: 314<br>>40: 80	-
Silva, 2009	Southeast, RJ	Cross-sectional	Women attending a private gynaecological clinic	100	300	33	14-79
Simões, 2017	Undefined	Cross-sectional (Abstract)	Women randomly selected	100	100	-	-
Smith, 2002	Southeast, SP	Case-control	Control group, women with newly diagnosed and not previously treated ICC and hospital-based controls	100	173	52.5	-
Soares, 2003	Northeast, AL	Cross-sectional	Sexually active women	100	341	34.4	15-63

Ströher, 2016	South, RS	Cross-sectional	Women of all ages and of various ethnic groups living in the city of Uruguaiana who were visiting Basic Health Units for routine gynecological examinations	100	51	42.7	16-86
Syrjänen, 2005	Multi, SP/RS	Cross-sectional/Prospective cohort	Consecutive series of women in their first visit to gynecology clinics	100	3351	NI	-
Tamegão-Lopes, 2014	North, PA	Cross-sectional	Women participating in a cervical cancer screening program	100	143	NI	-
Teixeira, 2016	South, RS	Cross-sectional	Mulheres em atendimento em ambulatórios de Ginecologia e Obstetrícia do Hospital Universitário Dr. Miguel Riet Corrêa Jr	100	200	33	-
Torres, 2018	North, AM	Cross-sectional	Women living in rural villages in proximity to Coari city	100	412	18-25: 79<br>26-36: 150<br>36-46: 99<br>46-54: 39<br>>=54: 45	-
Tota, 2016	Southeast, SP	Longitudinal	Population of low-income; women recruited from family medicine, gynecology, and family planning clinics in Sao Paulo, Brazil from 1993 to 1997	100	2462	32.7	18-57 (Sichero, 2017)
Trugilo, 2019	South, PR	Cross-sectional (case-control)	Women who underwent outpatient cytology testing between 2013 and 2015 at an ambulatory colposcopy facility of the Intermunicipal Consortium of Health of the Middle Paranapanema, at the University Hospital and Clinic Center of the State University of Londrina, and at two Basic Healthcare Units in Londrina—PR, Brazil.	100	349	-	-
Vieira, 2015	North, PA	Cross-sectional	Students undergoing routine cervical cancer screening	100	265	25	18-55
Waisberg, 2015	Southeast, SP	Prospective cohort	Women with and without Rheumatoid Arthritis (controls)	100	100	NI	18-74
Wohlmeister, 2016	South, RS	Cross-sectional	Women from one private medical unit	100	169	33	15-64
Xavier-Souza, 2018	Northeast, BA	Cross-sectional	Female adolescents attending a STI Referral Centre	100	789	16.08	10-19
Yamamoto, 2004	Southeast, SP	Cross-sectional	Women recruited from outpatient clinics and in-patient wards of public hospitals	100	196	52	18-79
**Penile region**							

Afonso, 2016	Southeast	Cross-sectional	30 penile foreskin specimens from men 18 to 56 years old who underwent circumcision and 110 penile swab samples were obtained from male patients 18 to 67 years old	0	140		18-67
Eleutério, 2010	Northeast, CE	Cross-sectional	Samples of penile scrapings received from private urological offices	0	88	-	-
Franceschi, 2002	Southeast, SP	Cross-sectional	Husbands or current stable partners of case and control women with ICC	0	109	-	-
Giraldo, 2008	Undefined	Cross-sectional	Asymptomatic men, partners of women with CIN	0	54	29	-
Menezes, 2014	Southeast, RJ	Cross-sectional	Asymptomatic men from STI clinic, dermatology clinic and a metallurgical factory	0	550	28.4	18-65
Rocha, 2012	Southeast, MG	Cross-sectional	Asymptomatic men whose sexual partner had presented HPV	0	43	-	18-60
Rocha, 2015	Southeast	Cross-sectional	Asymptomatic men, showing no clinically detectable HPV lesions	0	261	26.3	18-65
Rombaldi, 2006	South, RS	Cross-sectional	Male sexual partners of women with CIN	0	99	-	-
Silva, 2011	Southeast, SP	Prospective cohort	Control group, HIV negative patients	0	77	30.2	-
Sudenga, 2017b	Southeast	Cohort	Men aged 18-70 years living in Sao Paulo, Brazil (HIM Study)	0	1410	18-30: 242<br>31-44: 675<br>45-74: 178	-
**Anal region**							
Eleutério, 2015	Northeast, CE	Case control	Control group, women with colposcopy and HPV test negatives	100	62	32.3	-
Nyitray, 2010	Southeast, SP	Prospective cohort	HIM study, men from urogenital care and through general media advertising	0	902	-	18-70
Soares, 2011	North, PA	Cross-sectional	Control group, patients with non-neoplastic diseases of the anal canal	64	42	41.1	23-66
Sudenga, 2017a	Southeast, SP	Cohort	Men aged 18-70 years living in Sao Paulo, Brazil (HIM Study)	0	731	19-30: 290<br>31-44: 341<br>45-70: 100	18-70
Veo, 2015	Southeast, SP	Cross-sectional	Control group, women with normal colposcopy and colpocytology	100	102	44.8	-
**Oral region**							

Antunes, 2012	South, RS	Cross-sectional, abstract	Smokers and alcoholic patients	NI	37	-	-
Araujo, 2014	North, PA	Cross-sectional	Individuals without clinically diagnosable lesions	62	166	35.9	18-79
Cavenaghi, 2013	Southeast, SP	Cross-sectional	Populations from an assistance program in rural district	62.1	124	47.6	-
da Silva, 2007	Southeast, SP	Case-control	Control group who had no clinical evidence of disease and no history of cancer	NI	10	-	-
do Sacramento, 2006	Southeast, SP	Cross-sectional	Cancer-free individuals selected from hospital	52.1	50	28	16-52
Esquenazi, 2010	Southeast, RJ	Cross-sectional	Volunteers, young healthy adults	60	100	22.7	20-31
Goncalves, 2006	Southeast, SP	Cross-sectional	Women with genital HPV infection and healthy women	100	140	-	-
Horewicz, 2010	Southeast, SP	Cross-sectional	Good general health	NI	104	-	-
Kreimer, 2013	Southeast, SP	Cross-sectional from cohort study	HIM study, healthy men	0	499	NI	-
Machado, 2014	South, RS	Cross-sectional	Asymptomatic oral mucosa	0	559	23	18-68
Marques, 2015	Central-West, DF	Cross-sectional	Patients diagnosed with CIN and ICC, and their male partners	66	65	39	22-60
Oliveira, 2017	Southeast, RJ	Cross-sectional	Women who were referred to the Family Health Program from Niterói - None of the women had been vaccinated against HPV.	100	76	37	14-70
Peixoto, 2011	Northeast, BA	Cross-sectional	Women from outpatient clinics with hystopathologic diagnosis of genital HPV	100	100	30	20-40
Silva, 2016	Southeast, RJ	Cross-sectional (case-control)	HIV-positive individuals who attended at the University Hospital's ambulatory, Niterói City, Rio de Janeiro, Brazil, between 2009 and 2010. The control group included 120 volunteers from University Hospital's blood donors service, located in the same city	NI	197		18-75
Tristão, 2012	Undefined	Cross-sectional	Healthy patients	70	125	NI	17-54
Vidotti, 2014	Northeast, MA	Cross-sectional	Women with cervical lesions compatible with HPV infection	100	105	-	-

Xavier, 2009	Southeast, SP	Cross-sectional	Men attending the Clinic for Sexually Transmitted Diseases with HPV anogenital lesion(s)	0	30	29.3	18-56
Zonta, 2012	Southeast, SP	Cross-sectional	Incarcerated women with cervical lesions	100	27	-	18-60
**Genital region**							
Bomfim-Hyppólito, 2013	Northeast, CE	Cross-sectional	Participants from outpatient service	56.3	103	46.7	-
Soares, 2014	Southeast, SP	Cross-sectional	Men who had sex with men	0	658	NR	-
Stiepcich, 2012	Southeast, SP	Cross-sectional (abstract)	Patients of a private health institution	NI	15307	-	-
**More than one body regions analyzed**							
Afonso, 2013	Southeast, RJ	Cross-sectional	Women with CIN as well as their male sexual partners and asymptomatic couples	50	120	27.6 women<br>35.9 men	-
Autran, 2018	Northeast, CE	Cross-sectional	Immunocompetent women assisted at the medical clinic of Maternidade Escola Assis Chateaubriand, with or without genital intraepithelial lesions	100	20	-	-
Beder Ribeiro, 2014	Northeast, PE	Cross-sectional	Married couples, male partners had penile lesions	50	31	-	-
Carestiato, 2006	Southeast, RJ	Cross-sectional	Women for routine exams and men for medical suspicious of infections	79.7	7314	28.8	-
Castro, 2009	Northeast, AL	Cross-sectional	Women with genital HPV infection	100	30	-	-
Lima, 2014	Southeast, SP	Case control	Control group, HIV (-) women recruited from a sexual health clinic	100	100	-	-
Nicolau, 2005	Southeast, SP	Cross-sectional	Stable sexual partners of confirmed HPV-infected women	0	50	31.0	19-53
Rosenblatt, 2004	Undefined	Cross-sectional	Partners of women with CIN and normal women	33.3	180	NI	-

AL, Alagoas; AM, Amazonas; BA, Bahia; CE, Ceará; CIN, cervical intraepithelial neoplasia; DF, Distrito Federal; ES, Espírito Santo; GO, Goiás; HIM, Human Papilloma virus in Men; HIV, Human Immunodeficiency Virus; HPV, Human Papilloma virus; ICC, Invasive Cervical Cancer; MA, Maranhão; MG, Minas Gerais; MS, Mato Grosso do Sul; NI, Not Informed; PA, Pará; PB, Paraíba; PE, Pernambuco; PR, Paraná; RJ, Rio de Janeiro; RN, Rio Grande do Norte; RO, Rondônia; RS, Rio Grande do Sul; SC, Santa Catarina; SP, São Paulo; STI, sexually transmitted infection. *Age in urban area.
