# Supplementary material for: Prevalence of human papillomavirus (HPV) in Brazil: A systematic review and meta-analysis
Source: PLoS One. 2020 Feb 21;15(2):e0229154. doi: 10.1371/journal.pone.0229154 (PMC7034815; doi:10.1371/journal.pone.0229154)
Supplement: S2 Table — (PDF) [file pone.0229154.s004.pdf]

Additional File 3.

Assessment of studies quality

"Author, year"	Research question	Study population	Participation rate	Selection	Sample size	HPV measures	Population-based study
Afonso, 2013	Good	Good	Good	Good	Poor	Good	Poor
Afonso, 2016	Good	Good	Other	Other	Other	Good	Poor
Alvarez-Arguelles, 2009	Good	Poor	Poor	Poor	Poor	Good	Poor
Amaro-Filho, 2014	Good	Good	Other	Poor	Poor	Good	Poor
Amorim, 2017	Good	Good	Good	Other	Good	Good	Other
Antunes, 2012	Good	Poor	Poor	Poor	Poor	Good	Poor
Araujo, 2014	Good	Good	Other	Good	Good	Good	Poor
Araujo, 2014b	Good	Good	Other	Poor	Poor	Good	Poor
Augusto, 2014	Good	Good	Other	Good	Poor	Good	Poor
Autran, 2018	Good	Good	Other	Good	Other	Poor	Other
Ayres, 2017	Good	Good	Good	Good	Other	Good	Good
Baldi, 2015	Good	Good	Other	Good	Poor	Good	Poor
Baldin, 2011	Good	Good	Good	Good	Poor	Good	Poor
Batista, 2017	Good	Good	Good	Good	Good	Good	Good
Becker, 2001	Good	Good	Good	Good	Poor	Good	Poor
Bomfim-Hyppólito, 2013	Good	Good	Good	Good	Poor	Poor	Poor
Boon, 2001	Good	Poor	Other	Good	Poor	Good	Poor
Brito, 2002	Good	Poor	Good	Good	Poor	Good	Poor
Caixeta, 2015	Good	Good	Other	Good	Poor	Good	Poor
Campos, 2012	Good	Poor	Good	Poor	Good	Good	Poor
Campos, 2014	Good	Poor	Poor	Good	Poor	Good	Poor

Carestiato, 2006	Good	Poor	Other	Poor	Poor	Good	Poor
Cassel, 2014	Good	Good	Other	Good	Poor	Good	Poor
Castro, 2009	Good	Poor	Other	Good	Poor	Good	Poor
Cavenaghi, 2013	Good	Poor	Good	Good	Good	Good	Poor
Ceccato Junior, 2015	Good	Good	Other	Poor	Poor	Good	Poor
Chagas, 2015	Good	Good	Other	Poor	Poor	Good	Poor
Coser, 2013	Good	Good	Other	Good	Poor	Good	Poor
Coser, 2016	Good	Good	Other	Good	Other	Good	Good
Costa-Lira, 2017	Good	Good	Other	Good	Other	Good	Good
da Silva, 2007	Good	Poor	Other	Poor	Poor	Good	Poor
da Silva, 2012	Good	Good	Good	Good	Poor	Good	Poor
de Abreu, 2016	Good	Good	Other	Good	Poor	Good	Good
de Aguiar, 2014	Good	Good	Good	Poor	Good	Good	Good
de Almeida, 2014	Good	Poor	Good	Poor	Poor	Good	Poor
De Brot, 2017	Good	Good	Other	Good	Other	Good	Poor
de Mattos, 2011	Good	Good	Other	Poor	Poor	Good	Poor
de Medeiros, 2008	Good	Good	Other	Good	Poor	Good	Poor
de Oliveira, 2017	Good	Good	Other	Good	Other	Good	Good
do Sacramento, 2006	Good	Poor	Other	Poor	Poor	Good	Poor
dos Santos, 2013	Good	Good	Good	Poor	Poor	Good	Poor
Duarte, 2017	Good	Good	Other	Good	Other	Good	Good
Eleuterio, 2010	Good	Good	Other	Poor	Poor	Good	Poor
Eleuterio, 2015	Good	Good	Other	Poor	Poor	Good	Poor
Eluf-Neto, 1994	Good	Good	Good	Poor	Poor	Good	Poor
Esquenazi, 2010	Good	Good	Other	Poor	Poor	Good	Poor
Fedrizzi, 2008	Good	Good	Other	Poor	Poor	Good	Poor
Fedrizzi, 2009	Good	Poor	Other	Poor	Poor	Good	Poor

Fiqueiredo Alves, 2013	Good	Good	Other	Good	Good	Good	Good
Fonseca, 2015	Good	Good	Good	Good	Good	Good	poor
Franceschi, 2002	Good	Good	Good	Good	Poor	Good	Poor
Franco, 1995	Good	Good	Good	Good	Poor	Good	Poor
Freitas, 2009	Good	Poor	Good	Poor	Poor	Good	Poor
Gadelha, 2017	Good	Good	Other	Good	Other	Good	Good
Gillio-Tos, 2012	Good	Good	Other	Poor	Poor	Good	Poor
Giraldo, 2008	Good	Poor	Other	Poor	Poor	Good	Poor
Girianelli, 2010	Good	Good	Good	Good	Poor	Good	Poor
Golfetto, 2018	Good	Good	Poor	Good	Good	Good	Good
Goncalves, 2006	Good	Good	Other	Good	Poor	Good	Poor
Holanda, 2006	Good	Good	Other	Good	Good	Good	Poor
Horewikz, 2010	Good	Poor	Other	Poor	Poor	Good	Poor
Igansi, 2012	Good	Good	Other	Poor	Poor	Good	Poor
Krambeck, 2008	Good	Good	Other	Poor	Poor	Good	Poor
Kreimer, 2011	Good	Good	Other	Poor	Poor	Good	Poor
Kuil, 2017	Good	Good	Other	Good	Good	Good	Poor
Leite, 2018	Good	Good	Other	Good	Other	Good	Good
Lima, 2014	Good	Poor	Other	Good	Poor	Good	Poor
Lima, 2018	Good	Good	Other	Other	Other	Good	Other
Lippman, 2010	Good	Good	Good	Good	Poor	Good	Poor
Lopes, 2001	Good	Good	Good	Good	Poor	Good	Poor
Lorenzato, 2000	Good	Poor	Other	Poor	Poor	Good	Poor
Lorenzi, 2016	Good	Good	Other	Good	Poor	Good	Good
Lugo, 2018	Good	Good	Other	Good	Other	Good	Poor
Machado, 2014	Good	Poor	Other	Poor	Poor	Good	Poor
Marques, 2015	Good	Good	Poor	Poor	Poor	Good	Poor

Martins, 2012	Good	Good	Other	Good	Poor	Good	Poor
Martins, 2016	Good	Poor	Other	Poor	Other	Good	Poor
Martins, 2018	Good	Good	Good	Good	Other	Good	Poor
McCormick, 2015	Good	Good	Other	Good	Poor	Good	Poor
Menezes, 2014	Good	Good	Other	Poor	Poor	Good	Poor
Miranda, 2013	Good	Poor	Other	Good	Good	Good	Poor
Munoz, 1996	Good	Good	Other	Poor	Poor	Good	Poor
Naud, 2006	Good	Good	Good	Good	Poor	Good	Good
Nicolau, 2005	Good	Poor	Good	Good	Poor	Good	Poor
Nonato, 2016	Good	Good	Good	Good	Good	Good	Good
Nonnenmacher, 2002	Good	Poor	Good	Other	Good	Good	Poor
Noronha, 2011	Poor	Good	Good	Good	Poor	Poor	Poor
Nyitray, 2010	Good	Good	Good	Poor	Poor	Good	Poor
Oliveira, 2007	Good	Good	Good	Good	Other	Good	Good
Oliveira, 2010	Good	Poor	Good	Good	Poor	Good	Poor
Oliveira, 2013	Good	Good	Good	Poor	Good	Good	Poor
Oliveira, 2017	Good	Good	Other	Good	Other	Good	Poor
Padovani, 2013	Good	Good	Other	Poor	Poor	Good	Poor
Paesi, 2015	Good	Good	Other	Good	Good	Good	Other
Peixoto, 2011	Good	Poor	Other	Poor	Poor	Good	Poor
Peres, 2015	Good	Good	Other	Good	Other	Good	Good
Pinheiro, 2016	Good	Good	Other	Good	Other	Good	Good
Pinto, 2011	Good	Good	Good	Poor	Good	Good	Good
Porcari, 2018	Good	Good	Other	Other	Other	Good	Poor
Rama, 2006	Good	Good	Good	Poor	Poor	Good	Poor
Rama, 2010	Good	Good	Good	Good	Poor	Good	Poor
Rezende, 2014	Good	Poor	Other	Other	Poor	Good	Poor

Ribeiro, 2014	Good	Good	Other	Good	Poor	Good	Poor
Richardson, 2015	Good	Good	Other	Other	Other	Good	Other
Rocha, 2012	Good	Poor	Other	Poor	Poor	Good	Poor
Rocha, 2013	Good	Good	Good	Good	Good	Good	Good
Rocha, 2015	Good	Good	Good	Good	Good	Good	Good
Rodrigues, 2014	Good	Good	Good	Good	Other	Good	Good
Rodrigues, 2018	Good	Good	Other	Good	Good	Good	Good
Rombaldi, 2006	Good	Good	Good	Other	Poor	Good	Poor
Rosa, 2007	Good	Good	Other	Poor	Poor	Good	Poor
Rosenblatt, 2004	Good	Poor	Good	Other	Poor	Good	Poor
Roteli-Martins, 2011	Good	Poor	Good	Poor	Good	Good	Poor
Salcedo, 2015	Good	Poor	Good	Poor	Poor	Good	Poor
Santos, 2012	Good	Poor	Poor	Poor	Poor	Good	Poor
Santos, 2016	Good	Good	Other	Good	Other	Good	Good
Silva, 2009b	Poor	Good	Good	Poor	Poor	Good	Poor
Silva, 2011	Good	Good	Good	Good	Poor	Good	Poor
Silva, 2016	Good	Good	Other	Other	Other	Good	Other
Simões, 2017	Good	Poor	Other	Other	Other	Good	Other
Smith, 2002	Good	Good	Good	Good	Poor	Good	Poor
Soares, 2003	Good	Good	Good	Good	Other	Good	Good
Soares, 2011	Poor	Good	Other	Good	Poor	Good	Poor
Soares, 2014	Good	Good	Good	Good	Good	Good	Good
Stiepcich, 2012	Good	Poor	Other	Other	Poor	Poor	Poor
Ströher, 2016	Good	Good	Other	Good	Other	Good	Good
Sudenga, 2017a	Good	Good	Good	Good	Other	Good	Poor
Sudenga, 2017b	Good	Good	Good	Good	Other	Good	Good
Syrjänen, 2005	Good	Good	Other	Poor	Poor	Good	Good

Tamegão-Lopes, 2014	Good	Good	Other	Good	Poor	Good	Poor
Teixeira, 2016	Good	Good	Other	Good	Good	Good	Good
Torres, 2018	Good	Good	Other	Good	Other	Good	Good
Tota, 2016	Good	Good	Other	Good	Other	Good	Good
Tristão, 2012	Poor	Poor	Other	Other	Poor	Good	Poor
Trugilo, 2018	Good	Good	Other	Other	Other	Good	Other
Véo, 2015	Good	Good	Good	Good	Poor	Good	Poor
Vidotti, 2014	Good	Good	Good	Good	Good	Good	Poor
Vieira, 2015	Good	Good	Good	Good	Poor	Good	Poor
Waisberg, 2015	Good	Poor	Good	Poor	Poor	Good	Poor
Wohlmeister, 2016	Good	Good	Other	Good	Other	Good	Poor
Xavier, 2009	Good	Poor	Other	Good	Poor	Good	Poor
Xavier-Souza, 2018	Good	Poor	Other	Other	Other	Other	Good
Yamamoto, 2004	Good	Poor	Good	Good	Poor	Good	Poor
Zonta, 2012	Good	Good	Good	Good	Poor	Good	Poor

Questions: Was the research question or objective in this paper clearly stated?; Was the study population clearly specified and defined?; Was the participation rate of eligible persons at least 50%?; Were all the subjects selected or recruited from the same or similar populations?; Was a sample size justification, power description, or variance and effect estimates provided?; Were the HPV measures (dependent variables) clearly defined?; Was the study a population-based study?

Evidence profile for prevalence of HPV in Brazil (GRADE)

**Outcome**	**Study design**	**N studies; N participants**	**Quality of evidence assessment**						**Effect**
			**Risk of bias**	**Inconsistency**	**Indirectness**	**Imprecision**	**Other**	**Level of evidence**	**Overall prevalence of HPV**
Cervical region	Observational	105 studies; 50,822 participants	Serious[1]	Not serious[2]	Not serious	Not serious	None	Low +OO	25.41% (22.71, 28.32)
Oral region	Observational	20 studies; 2,494 participants	Serious[1]	Serious[3]	Serious[4]	Not serious	None	Very Low +OOO	11.89% (6.26, 21.43)
Penile region	Observational	12 studies; 2,956 participants	Serious[1]	Serious[3]	Serious[4]	Not serious	None	Very Low +OOO	36.21% (23.40, 51.33)
Anal region	Observational	7 studies; 1,272 participants	Serious[1]	Serious[3]	Serious[4]	Not serious	None	Very Low +OOO	25.68% (14.64, 41.04)

[1] Most studies evaluated selected population (few studies with community-based samples).

[2] Although statistical inconsistency varies widely, point estimates across different regions remain similar.

[3] Prevalence varies widely across different regions, with low number of participants in Northeast, North and Central-West, especially for body sites other than cervical.

[4] Most studies from Southeast region; other regions not represented or underrepresented.
