# Supplementary figures and images for: Prevalence of human papillomavirus (HPV) in Brazil: A systematic review and meta-analysis
Source: PLoS One. 2020 Feb 21;15(2):e0229154. doi: 10.1371/journal.pone.0229154 (PMC7034815; doi:10.1371/journal.pone.0229154)

(a) cervical

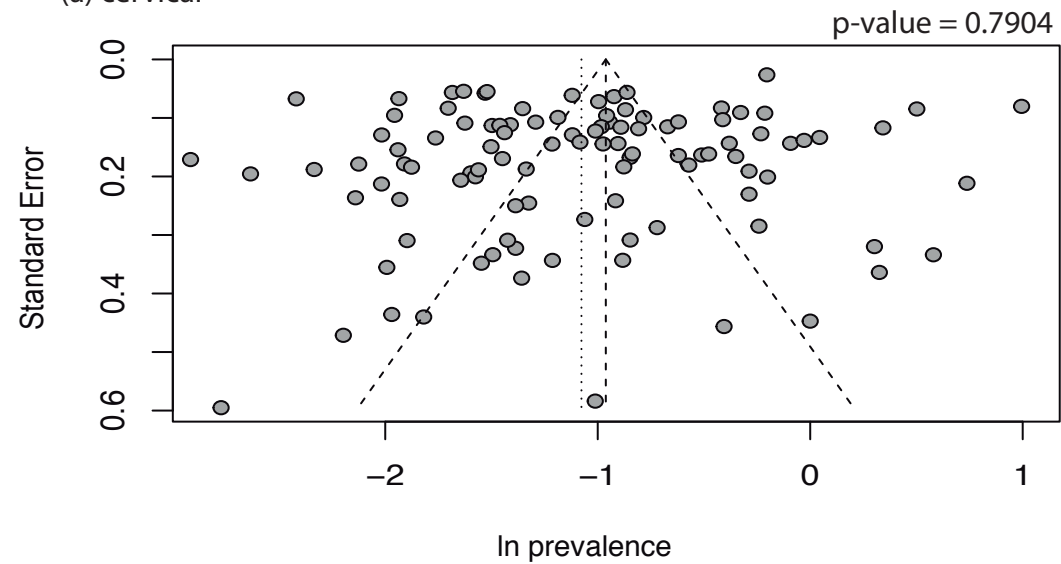

(b) penile

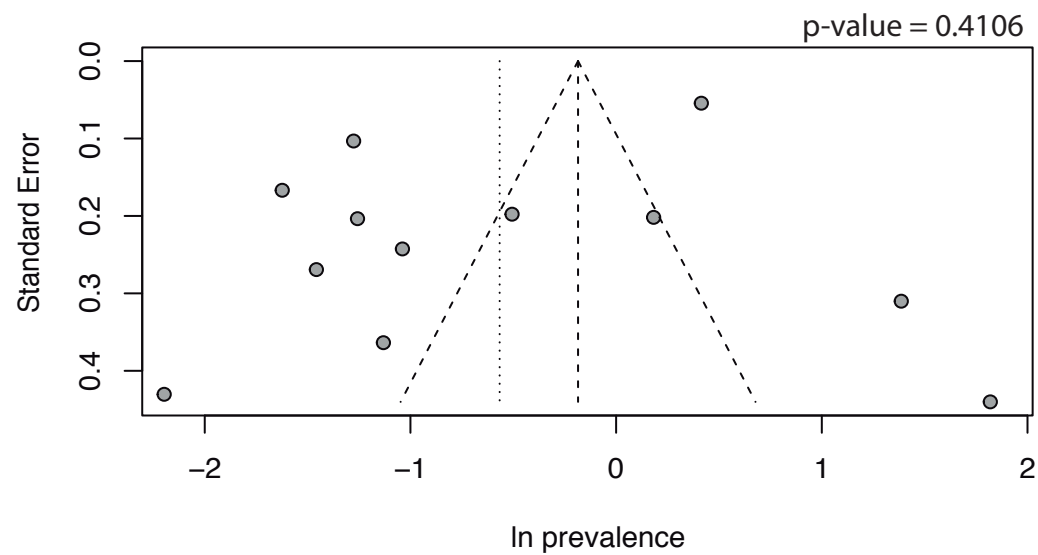

(c) anal

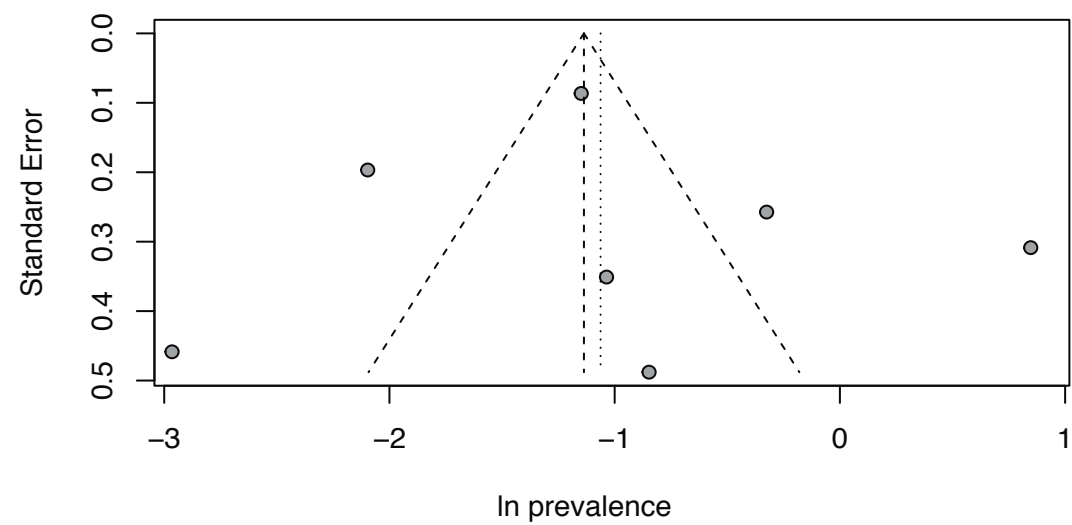

(d) oral

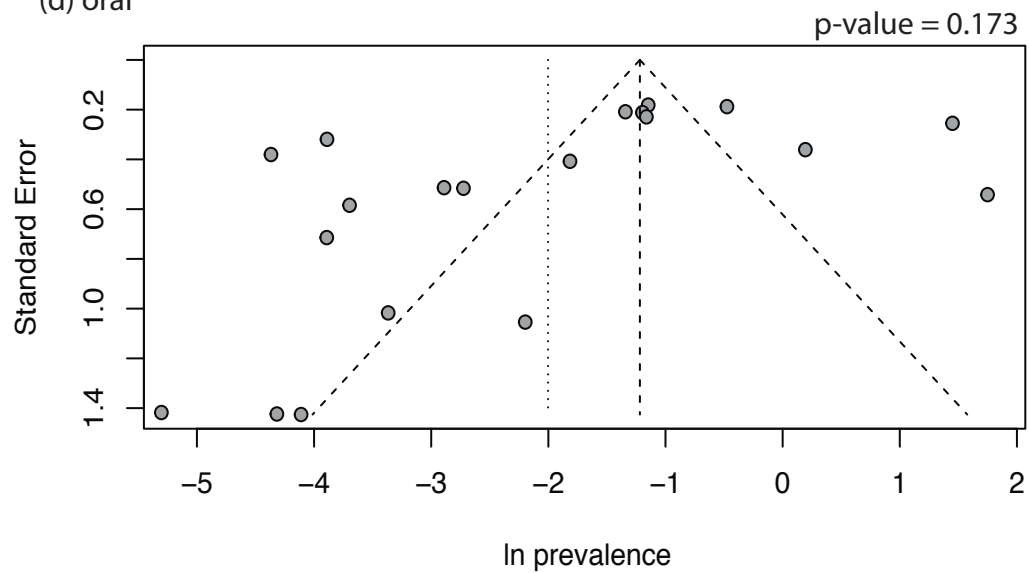

Supplement: S1 Fig — (PDF) [file pone.0229154.s005.pdf]

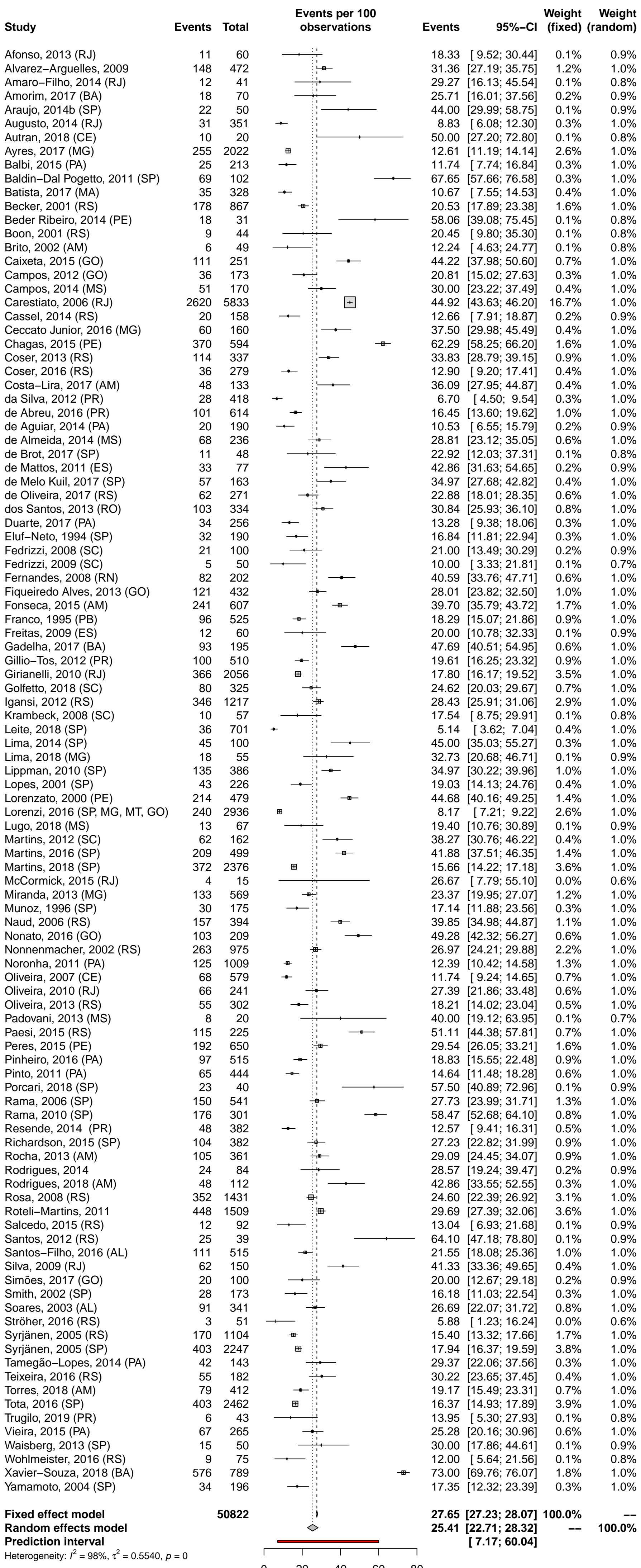

Supplement: S2 Fig — (PDF) [file pone.0229154.s006.pdf]

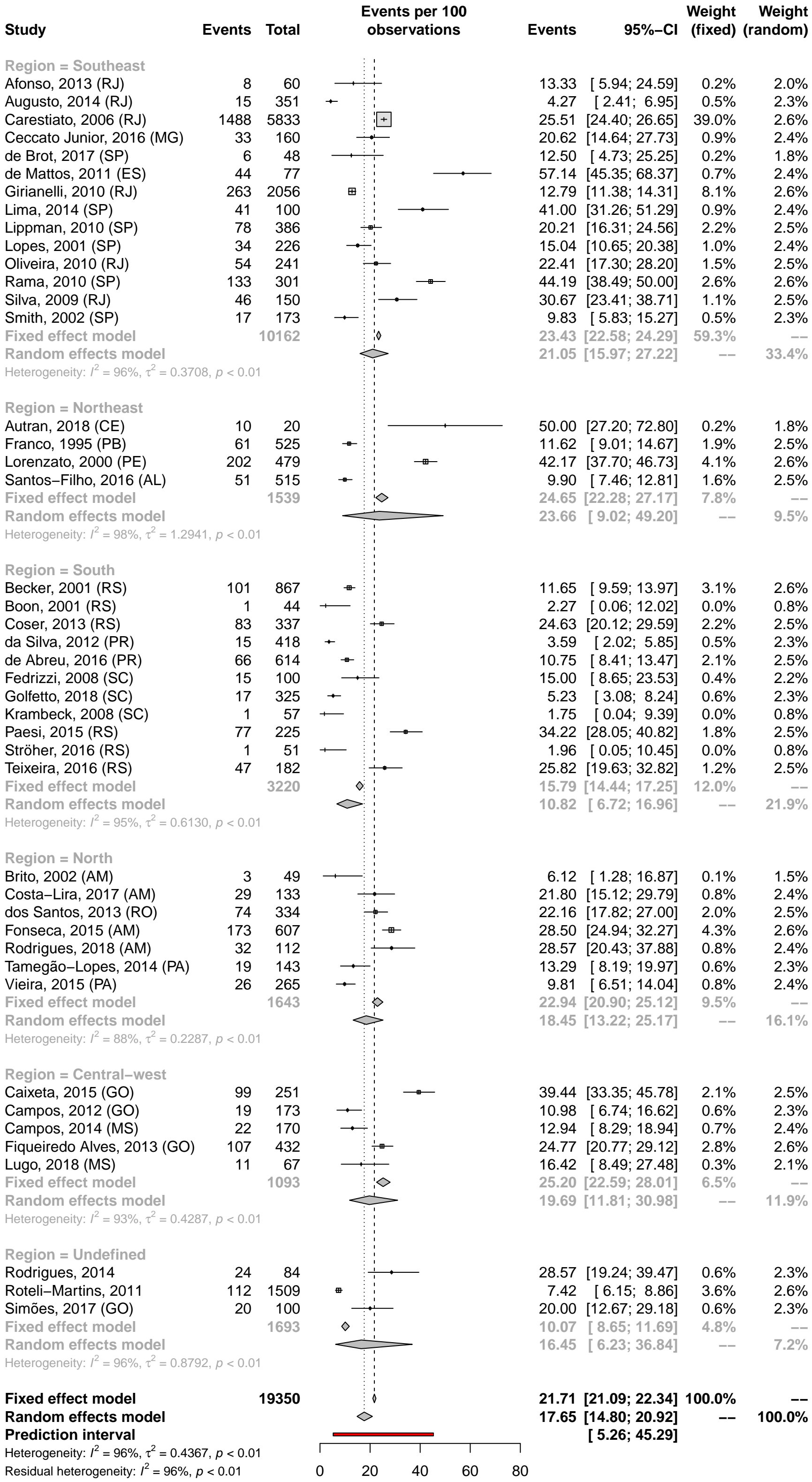

Supplement: S3 Fig — (PDF) [file pone.0229154.s007.pdf]
